# Strategies to support the mental health and well-being of health and care workforce: a rapid review of reviews

**DOI:** 10.3389/fmed.2025.1530287

**Published:** 2025-03-19

**Authors:** Cindy E. Frias, Nimesh Samarasinghe, Cecilia Cuzco, Jaseem Koorankot, Andrés de Juan, Husameldin Mohamed Ali Rudwan, Hanan F. Abdul Rahim, Adela Zabalegui, Iain Tulley, Sanaa T. Al-Harahsheh, Mona Shaheen S. T. Al-Homaiddi, Meredith Fendt-Newlin, Jim Campbell

**Affiliations:** ^1^Hospital Clinic of Barcelona, Barcelona, Spain; ^2^Hamad Medical Corporation, Doha, Qatar; ^3^Fundamentals and Clinical Nursing Department, University of Barcelona, Barcelona, Spain; ^4^College of Health Sciences, Qatar University, Doha, Qatar; ^5^Qatar Foundation, Doha, Qatar; ^6^World Health Organization, Geneva, Switzerland

**Keywords:** public health workforce, rapid review, healthcare workers, mental health, burnout, depression, anxiety, health systems resilience

## Abstract

**Background:**

Countries globally face challenges in educating, employing and retaining their health and care workforce, largely due to underinvestment in health systems. Health and care workforce report significantly greater levels of job-related burnout and mental health problems, which in turn are associated with poorer patient outcomes, increased medical errors, diminished quality and safety, decreased patient satisfaction, and reduced healthcare efficiency.

**Objective:**

We conducted a rapid review of systematic reviews to evaluate the mental health and well-being of health and care workers since the onset of the COVID-19 pandemic and to identify interventions available at organizational and individual levels.

**Methods:**

PubMed and Epistemonikos were searched for systematic reviews published between May 2022 and February 2024. The inclusion criteria were systematic reviews written in English with quantitative design, with or without meta-analysis.

**Results:**

Fifty articles met the criteria for inclusion in the analysis. Overall, there has not been a significant change in the prevalence of depression and anxiety among health and care workforce since 2022, suggesting the impact of the COVID-19 pandemic on workers’ mental health and well-being was not specific to their experience working during the pandemic. Sixteen studies reported two types of mental health and well-being interventions: individual-level interventions and organizational-level interventions with specific impact on mental health and work environment variables. No specific policy interventions were found. However, some studies suggested policy interventions to improve the mental health and well-being of the health and care workforce.

**Discussion:**

Our analysis highlighted the need for systemic changes to protect the mental health and well-being of the health and care workforce in the post-COVID-19 era. Despite the wealth of evidence on mental health problems and on effective interventions, there remains a notable gap in systemic implementation and organizational accountability. The call to action for a paradigm shift must be embraced and we must strive to build resilient healthcare systems and invest in active support and sustain them, incorporating structural, non-structural and functional aspects of organizational resilience.

## 1 Introduction

Countries worldwide face significant challenges in educating, employing, and retaining their health and care workforce, largely due to underinvestment in health systems. These challenges are particularly pronounced in low-income countries and among the 55 countries listed on the WHO Support and Safeguards List 2023 (1). Where shortages, low pay, unsafe working conditions, and high-stress levels persist (2). These systemic issues have led to a growing mental health burden among health and care workers (HCWs), exacerbating burnout, absenteeism, and reduced job satisfaction (2, 3). The resulting impacts extend beyond individual workers, contributing to poorer patient outcomes, decreased healthcare efficiency, and widening gaps in healthcare delivery (3, 4).

The World Health Organization (WHO) defines HCWs as individuals engaged in work actions intended to improve health, including doctors, nurses, midwives, public health professionals, community health workers, and traditional medicine practitioners (4). Mental health, defined by the WHO as a state of well-being that enables individuals to cope with life stresses and contribute to their communities, is a critical component of overall health and a fundamental human right (5, 6). Similarly, well-being, encompassing quality of life and societal resilience, is influenced by social, economic, and environmental conditions (7). These definitions highlight the urgent need to address mental health and well-being as central components of workforce sustainability (7, 8).

The COVID-19 pandemic exposed and intensified preexisting vulnerabilities within healthcare systems, placing HCWs under immense pressure. During this period, HCWs experienced increased workloads, emotionally charged situations, and stigma surrounding mental health care, resulting in heightened levels of anxiety, depression, and burnout (9, 10). These challenges highlighted the inadequacy of organizational support structures and the need for systemic changes to safeguard HCWs' mental health and well-being (10–14).Building on the recommendations “Our Duty of Care: A global call to action to protect the mental health of health and care workers,” (15) this rapid review evaluates the mental health and well-being of HCWs since the onset of the COVID-19 pandemic. It is further informed by World Health Assembly (WHA) Resolution 74.14 (16) which called for the development of the Global Compact for Health and Care Workers to alleviate mental health disorders and improve HCW well-being. The review also draws on other WHO guidelines on mental health at work to identify supportive interventions and programs. Specifically, it examines individual and organizational level interventions aimed at addressing these challenges. Despite the wealth of evidence and guidance, the systemic implementation of such measures remains limited. By synthesizing recent findings, this review seeks to address this gap and propose strategies to safeguard HCWs’ mental health and build resilience within healthcare systems (11, 17).

Therefore, this rapid review aims to evaluate the mental health and well-being of health and care workers since the onset of the COVID-19 pandemic and to identify interventions available at organizational and individual levels.

## 2 Materials and methods

We conducted this Rapid Review using Preferred Reporting Items for Systematic Review and Meta-analysis (PRISMA) guidelines (18). Two authors independently conducted the literature search, study selection, and data extraction. Any disagreements were resolved by discussion with a third author when necessary.

### 2.1 Study search

The literature search utilized two electronic databases PubMed and Epistemonikos. Both were also used in the 2022 Our Duty of Care report (15). The latter database focuses on systematic reviews and regularly updates from several databases including the Cochrane Database of Systematic Reviews (CDSR), EMBASE, and PsycINFO. The search strategy used for both databases was partially based on the previous Our Duty of Care report. For PubMed database, the following search strategy was implemented: (“Mental Health”[Mesh] OR “Anxiety”[Mesh] OR “Depression”[Mesh] OR Burnout OR Distress OR Stress) AND (“Nurses”[Mesh]) OR “Physicians”[Mesh] OR Doctors OR Health Care Workers OR Health Workers)] NOT (Pati* OR Qualitative OR Scoping OR Synthesis). For Epistemonikos database, the following search strategy was used with some minor changes respecting the other: Mental Health OR Anxiety OR Depression OR Burnout OR Distress OR Stress AND Nurses OR Physicians OR Doctors OR Health Care Workers OR Health Workers NOT Pati* NOT Qualitative NOT Scoping NOT Synthesis. The search was limited from the period of May 2022 (which was the final period carried out for the Our Duty of Care Report, 2022) to February 2024. The terms chosen for both databases can be seen in Supplementary material S1.

### 2.2 Inclusion criteria

The inclusion criteria chosen were nearly identical to those used in the previous review 2 years ago to ensure consistency in addressing the same area. However, for this review, the articles did not need to measure the impact of COVID-19 specifically, as many recent studies no longer consider it a primary focus. Inclusion criteria were English-language, quantitative systematic reviews with or without meta-analysis, available in full text and published between May 2022 and February 2024. Additionally, articles were required to include a mental health outcome, including burnout or stress, anxiety, depression, suicidal ideation, trauma, insomnia, and/or sleep disturbances. Moreover, the selected articles focused on or included the health and care workforce, such as physicians, nurses, community health workers, physical therapists, pharmacists, and other related professions. All these criteria were followed based on the duty of care report protocol.

### 2.3 Exclusion criteria

The exclusion criteria were as follows: studies including healthcare students or the general population with no separate analysis of the health and care workforce, publications other than systematic reviews or with qualitative or mixed methods designs, studies written in languages other than English and studies that did not measure or did not track any mental health related outcome such as those specified in the inclusion criteria.

### 2.4 Study selection

**Figure 1:**
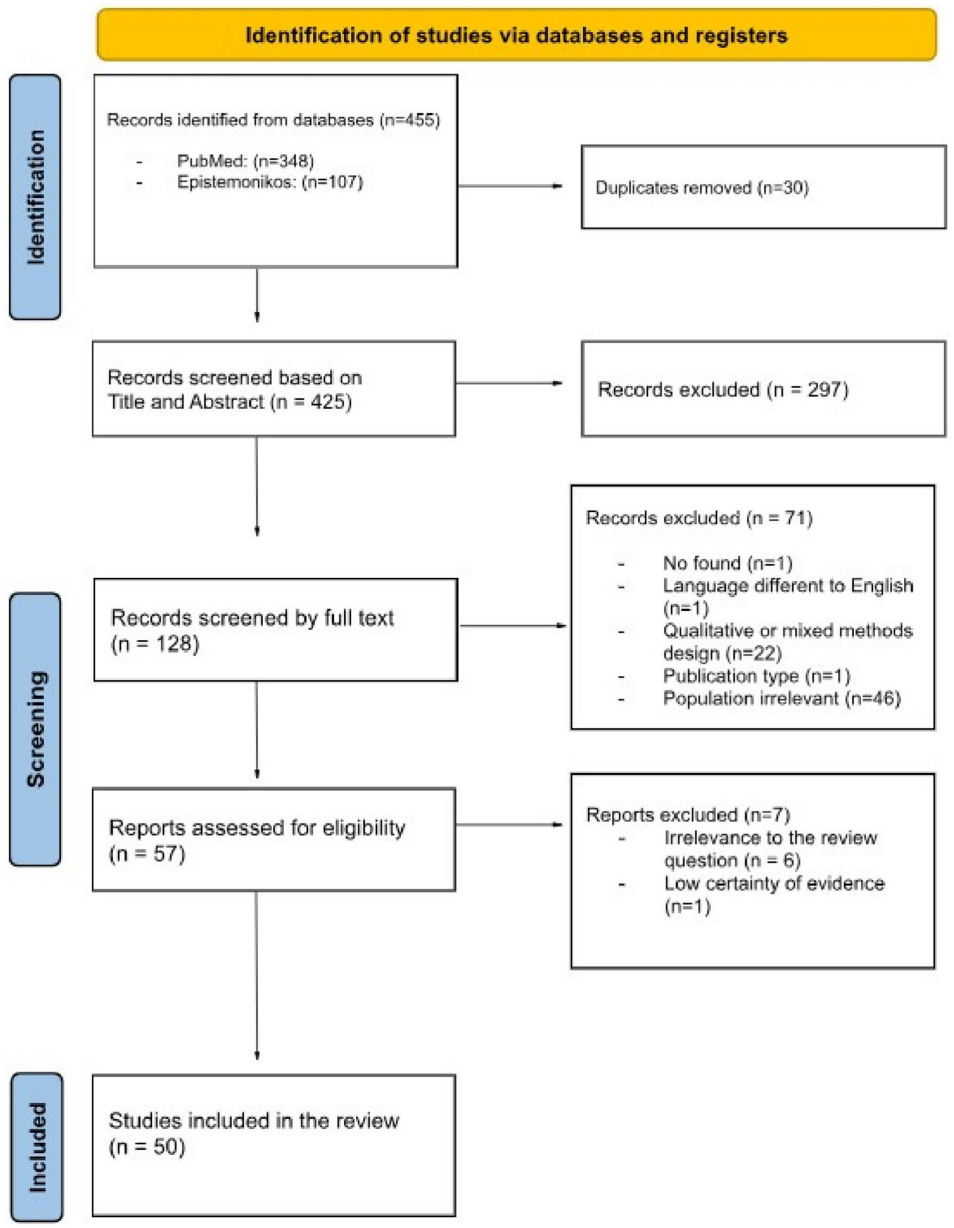
PRISMA flowchart of the study selection process.

In the first screening process, two independent researchers (AD and CC) screened the titles and abstracts of the searched documents from both databases to identify potentially related articles meeting the eligibility criteria. To remove duplicates, the Zotero^®^ and Rayyan^®^ platforms were used. The titles and abstracts were initially read, according to the pre-established criteria, by two independent reviewers; besides, the same researchers independently reviewed the selected full texts. The final inclusion of studies was decided through the two-step screening process. In the screening process, discrepancies between the researchers were resolved through discussion with a third researcher (CF). The study selection process is presented following a PRISMA flow diagram in Figure 1.

### 2.5 Risk of bias assessment

The current review did not conduct a formal risk of bias or quality assessment, which is often omitted in rapid reviews. Due to the limited time available for data analysis and considering that all included articles were systematic reviews—most of which had already assessed the methodological quality of the studies within them—no additional specific risk of bias assessment was conducted. However, we ensured that all included studies had undergone a prior risk of bias evaluation, thereby enhancing the reliability and robustness of the evidence.

### 2.6 Data extraction

Data extraction was conducted independently by two reviewers (AD and CC) to ensure accuracy and consistency. Any conflicts were resolved by a third reviewer (CF). To ensure the integrity of the assessment, we piloted the data extraction form on three studies. The extracted data included publication date, author(s), the title of the study, study design, country or geographic area covered, total population size, types of health and care workforce included in the study, an assessment tool to measure mental health burden and well-being, and the aim of studies related to mental health variables and well-being.

#### 2.6.1 Data analysis

To conduct a detailed and comprehensive analysis of the results of each included study, a description table was designed to extract all relevant data related to the main outcomes related to mental health burden, well-being, and work environment. In addition, for each included article, we analyzed whether the interventions aimed at improving health and care workers’ mental health or well-being had been designed for implementation at an organizational or individual level and the benefits obtained on mental health, well-being, and the work environment. Finally, each included study was also analyzed to see if recommendations at the government level based on actions at the policy level were presented.

## 3 Results

### 3.1 Study search

The flowchart of the study selection process is shown in Figure 1. The initial search found 455 references. After removing duplicates, 425 records were screened by title and abstract. Then, 128 full-text articles were retrieved for detailed evaluation. After review, 71 articles were removed for the following reasons: wrong study design (i.e., qualitative design only or qualitative design combined with other design) (*N* = 22), wrong population (*N* = 46), and other reasons (*N* = 3). “Wrong population” refers to studies on students or the general population instead of health and care workers. “Other reasons” include studies not being in English, wrong publication type, or not being found. Fifty seven studies were assessed for eligibility, leading to the exclusion of 7 studies. Fifty studies were finally included. Therefore, the intervention of the third reviewer was not necessary.

### 3.2 Characteristics of the articles included

An overview of the 50 studies selected for data extraction and analysis is presented in Supplementary Table 1. Fifty studies were included, of which 34 included a meta-analysis, and 16 were systematic reviews without meta-analyses. The studies were conducted in China (*n* = 19), France (*n* = 5), England (*n* = 4), Korea (*n* = 4), and Brazil (*n* = 3). One study was conducted in each of the following countries: Canada, Greece, South Africa, India, Lebanon, Saudi Arabia, Singapore, Finland, USA, Romania, Spain, Hungary, Indonesia, and Australia.

### 3.3 Participants

In the studies, the sample sizes ranged from 29 HCWs (19) to more than 341,014 (20) HCWs. Samples were predominantly female (at least more than 50% in 14 of the included studies). Most of the participants were nurses and doctors. Some studies also included the participation of midwives (21), occupational therapists, speech therapists (22), social workers (23), dentists (24, 25), technicians and administrative staff (26), paramedics, laboratory and X-ray technicians (19, 20, 23, 27–29).

### 3.4 Aim of the articles included

The studies measured mental health outcomes, including anxiety, depression, burden, and Post Traumatic Stress Disorder (PTSD), and well-being outcomes, such as job stress and satisfaction, sleep disorders, quality of life (QoL), general health and job stalking (job stalking is described as unwanted harassing behavior towards health and care workforce from patients or even from patients’ relatives). It is undesirable and repeated behavior, including following or monitoring someone, writing letters, calling by phone, communicating on the Internet, giving unsolicited gifts, and making annoying requests (30) and presenteeism. Likewise, some results associated with the work environment, such as workplace bullying, were evaluated.

### 3.5 Main results

#### 3.5.1 The mental health and well-being of HCWs since the COVID-19 pandemic

The updated prevalence estimates for anxiety were lower than for the general population, especially for men and women (28) until study estimates ranged from 6 to 90% (20). The level of depression symptoms was also lower than for the general population (28) rates varied until 91.30% (M = 27.23%, M = 23.28%) (23).

HCW reported that burnout and moral distress were conditions that had been present since before the pandemic, but that worsened due to the pressure of care at that time. This worsening was more evident in specific health and care groups, such as nurses and doctors. However, no significant differences have been observed compared to the previous report’s data, even if it has been noted that Intensive Care Unit (ICU) doctors and nurses exhibit higher levels of burnout, with 42 and 45%, respectively. An analysis of 20,723 ICU professionals revealed that the prevalence of high burnout levels did not differ significantly (*p* = 0.63) between ICU physicians (0.41 [95% CI, 0.33; 0.5]) and ICU nurses (0.44 [95% CI, 0.34; 0.55]) However, the proportion of ICU professionals with a high level of emotional exhaustion was higher in ICU nurses than in ICU physicians (0.42 [95% CI, 0.37; 0.48] and 0.28 [0.2; 0.39], respectively, *p* = 0.022) (31). We found burnout prevalence rates until 95% (32). A meta-regression analysis showed that the association between workplace bullying, and job burnout was stronger in studies with a higher percentage of females (coefficient beta = 0.01, 95% CI = 0.006 to 0.019, *p* = 0.001) (33). Several studies have examined the relationship between sociodemographic variables and burnout. Findings indicate that gender is not a significant predictor of burnout. However, educational attainment demonstrates a significant association, with nurses holding bachelor’s degrees exhibiting the highest levels of burnout among all academic groups (34).

The overall prevalence of PTSD ranged between 14% and 16% during the pandemic, 19% between 1 and 6 months after the end of the pandemic, then decreased to 8% over a year later. According to the results of our rapid review the prevalence of PTSD was lower after the pandemic ended and tended to be lower after the following year (22). However, it is noted that alterations in the sleep–wake cycle pattern are still present in HCWs. Some authors also found a high level of sleep disturbance with a prevalence of insomnia (46.9, 95% CI: 31.8, 62.5%; I2 = 97.7%, *p* < 0.001) (35).

A revision showed the prevalence of PTSD was 65.9% (IC 95%: 62.6%, 69%; I2=0%, *p* < 0.001) (35).Other mental health problems were found: 18.8% of men and 10.7% of women used alcohol to cope. 22.8% reported a lifetime suicidal ideation, with 10.4% reporting serious suicidal ideation and 3.1% reporting having previously attempted suicide. No gender difference was found, but there was a higher prevalence of women who ‘wished they were dead’ (28). One study found the rate of suicidal thoughts to be 1.5 to 3 times higher than the national average, but only 26% of those with suicidal thoughts had sought help, and 60% of those with suicidal ideation were hesitant to seek help due to concerns about their career (36). A review revealed that the rates of fear occurrence were (52.1, 95% CI: 30.1, 73.3%; I2 = 98.1%, *p* < 0.001) (35) (Supplementary Table 2). Supplementary Table 3 describes the prevalence of mental health disorders by region and occupation.

#### 3.5.2 Evidence of interventions

This rapid review also considered studies that provided interventions that addressed outcomes related to the mental health and well-being of HCWs, in any clinical care setting where health and care workforce provide care.

Of the 50 studies included, 16 studies reported the outcomes of interventions on health and care workforce’s mental health and well-being. After analyzing each one of them, they were classified into two types of interventions: interventions at the individual level and at the organizational level. These types of interventions or categories are based on the WHO guidelines on mental health at work. In these guidelines, the WHO provides evidence-based global public health guidance on organizational and individual interventions for the promotion of positive mental health and the prevention of mental health conditions, as well as recommendations on returning to work after absence associated with mental health conditions and obtaining employment for people living with mental health conditions (37).

##### 3.5.2.1 Individual-level interventions

In the included reviews, various interventions were carried out at the individual level, such as single interventions with one or several sessions, multi-component interventions, and interventions focused on alleviating mental health symptoms to also improve emotional well-being.

- Interventions with one or more sessions*: Well-being Centers*: These centers were equipped with staff (‘well-being buddies’) trained to offer psychological first aid to personnel of an acute hospital trust (listening, comforting and directing towards services, as needed) (38). *Training Programs*: Simulation-based teamwork training to develop leadership and communication skills, crucial during crises (38). *Resilience Training Program* (“R2 for Leaders”): Comprising virtual sessions to equip healthcare leaders with skills for organizational leadership and staff support (38). *Psychoeducation programs* showed a significant impact on stress reduction and how nurses cope with challenges using positive coping mechanisms (39).
- Multi-component interventions: *Specific Multi-Component Programs*: Workplace recognition; infection protection measures, reasonable work shift arrangements, logistical support, reorganization of wards and increased nurse-to-patient ratios; training on PPE use and its availability; establishment of a psychological help desk, promotion of autonomy among nurses, limiting work hours, adjusting staffing levels, providing information updates, offering immune-boosting supplements, and mental health support services. The multi-component prevention programs demonstrate potential protective effects, such as reducing anxiety and depression, and enhancing the quality of the psychosocial work environment, including job control, managerial and peer support, and workplace relationships. However, the confidence in these findings is low due to reliance on observational study designs and significant risks of selection and confounding bias (38).
- Interventions focused on alleviating specific symptoms: *Mindfulness-based interventions (MBI)*: focused on stress reduction using relaxation, yoga, breathing, and physical exercise. The evidence suggests that MBI training moderately reduces stress, but it shows no significant effect on anxiety (40). However, some authors concluded that MBI appears to alleviate stress and depression and has beneficial effects on the well-being of nurses (21, 41–43). Yang et al., also confirmed that MBI may be effective in reducing the symptoms of anxiety, depression, and stress. In addition, the training effectively reduces burnout (29, 40).

**Figure 2:**
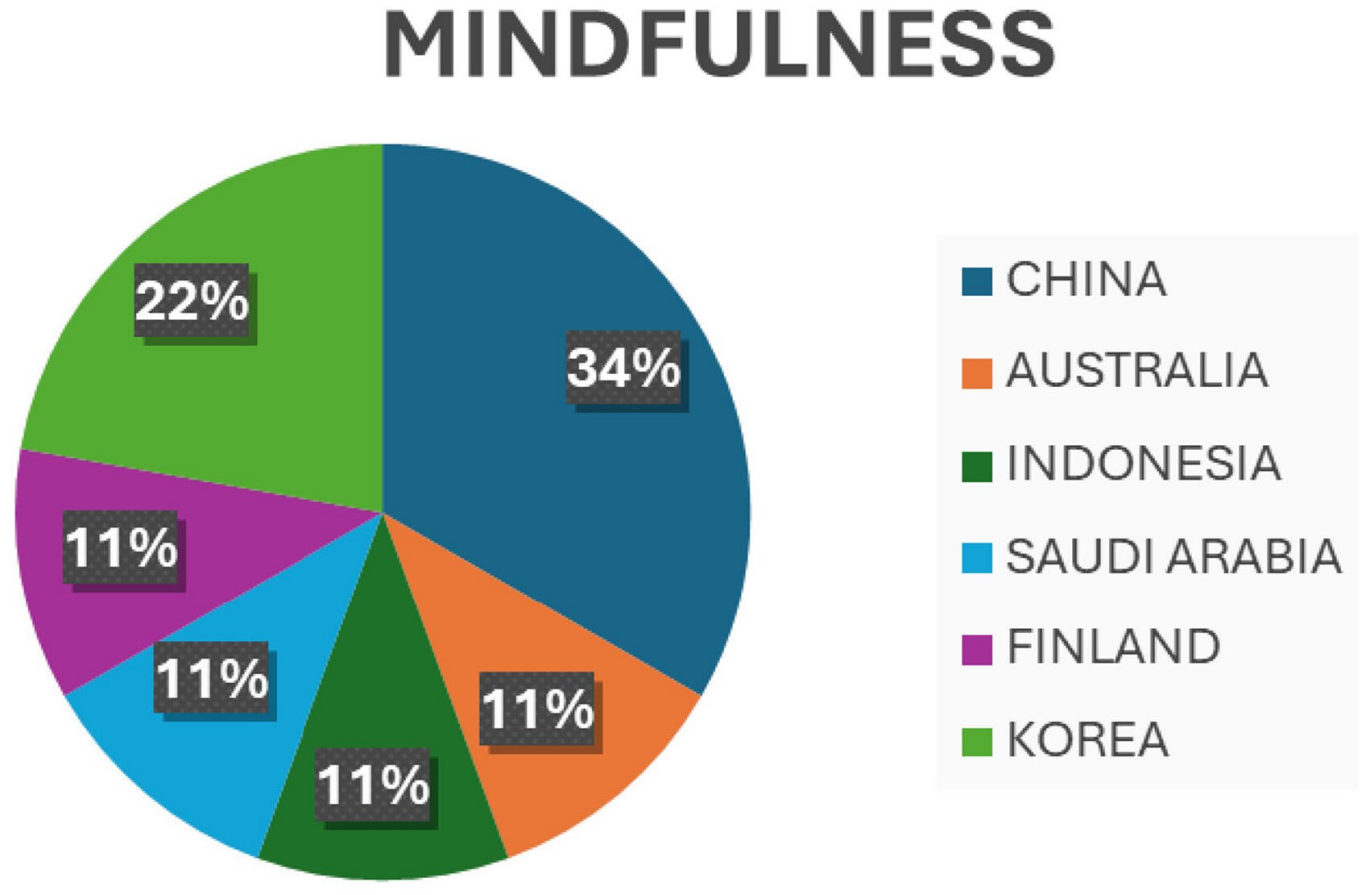
Percentage of individual mindfulness interventions by region.

**Figure 3:**
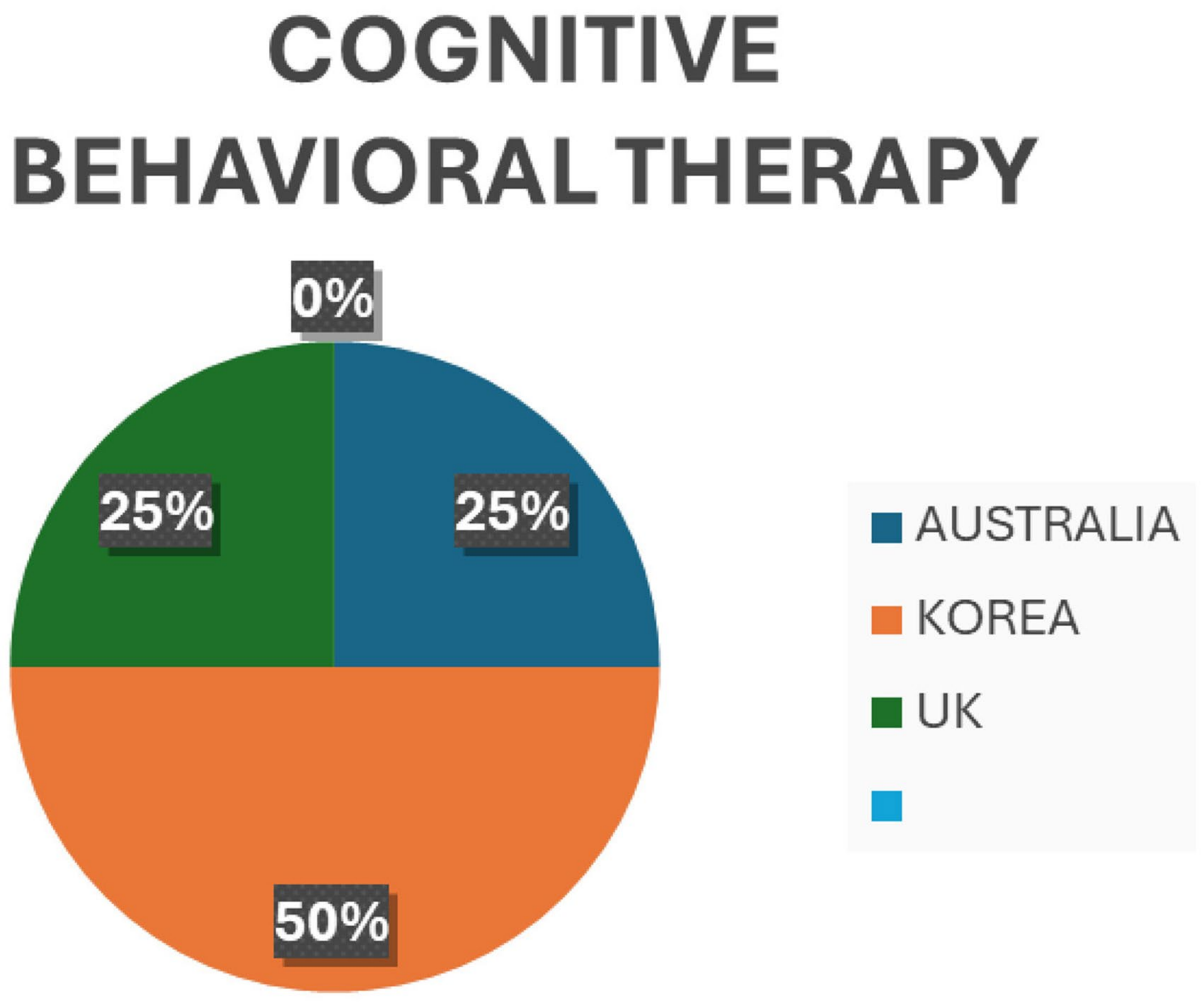
Percentage of cognitive behavioral therapy at individual level by region.

**Figure 4:**
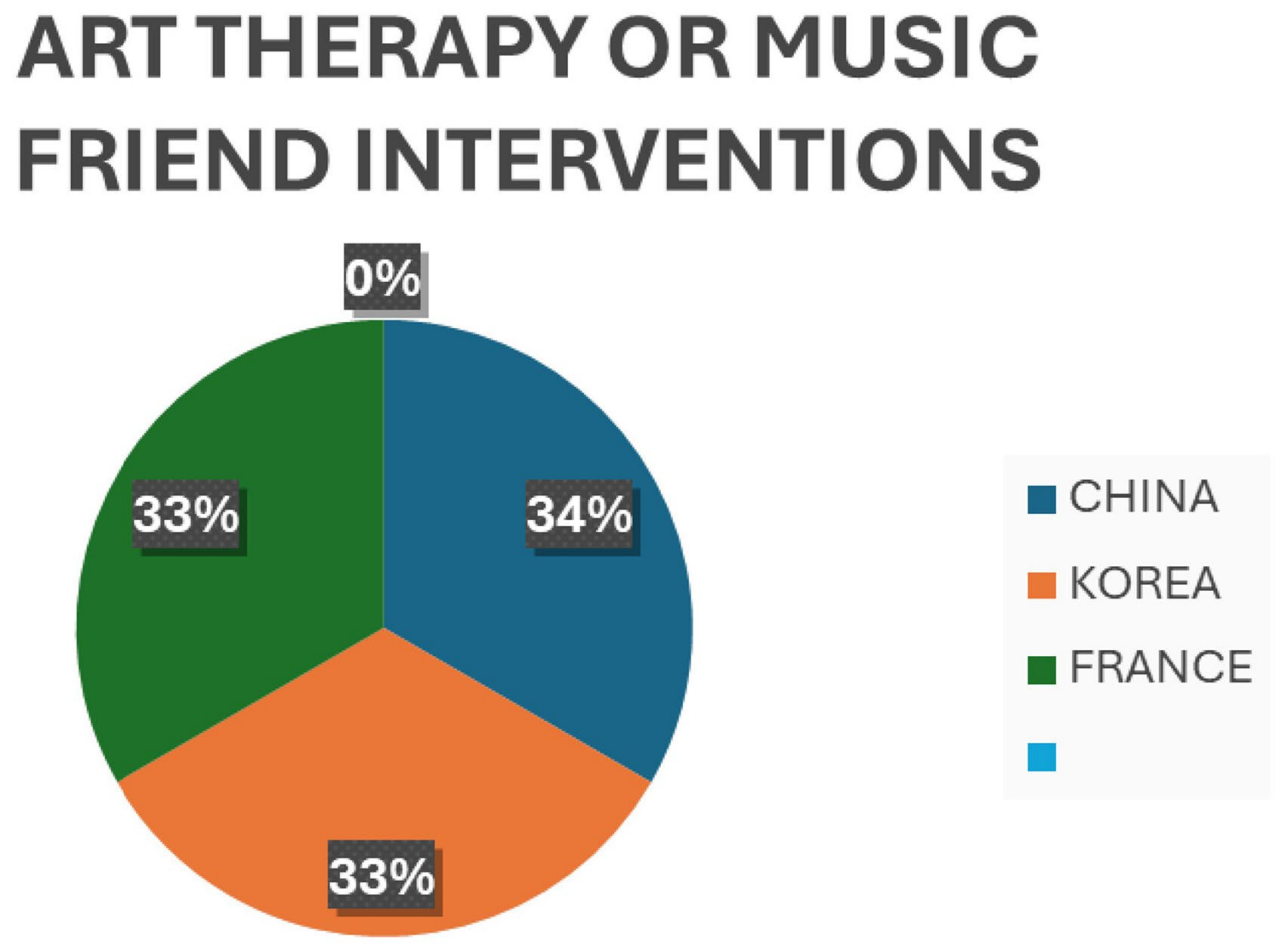
Percentage of art therapy/music intervention at individual level by region.

**Figure 5:**
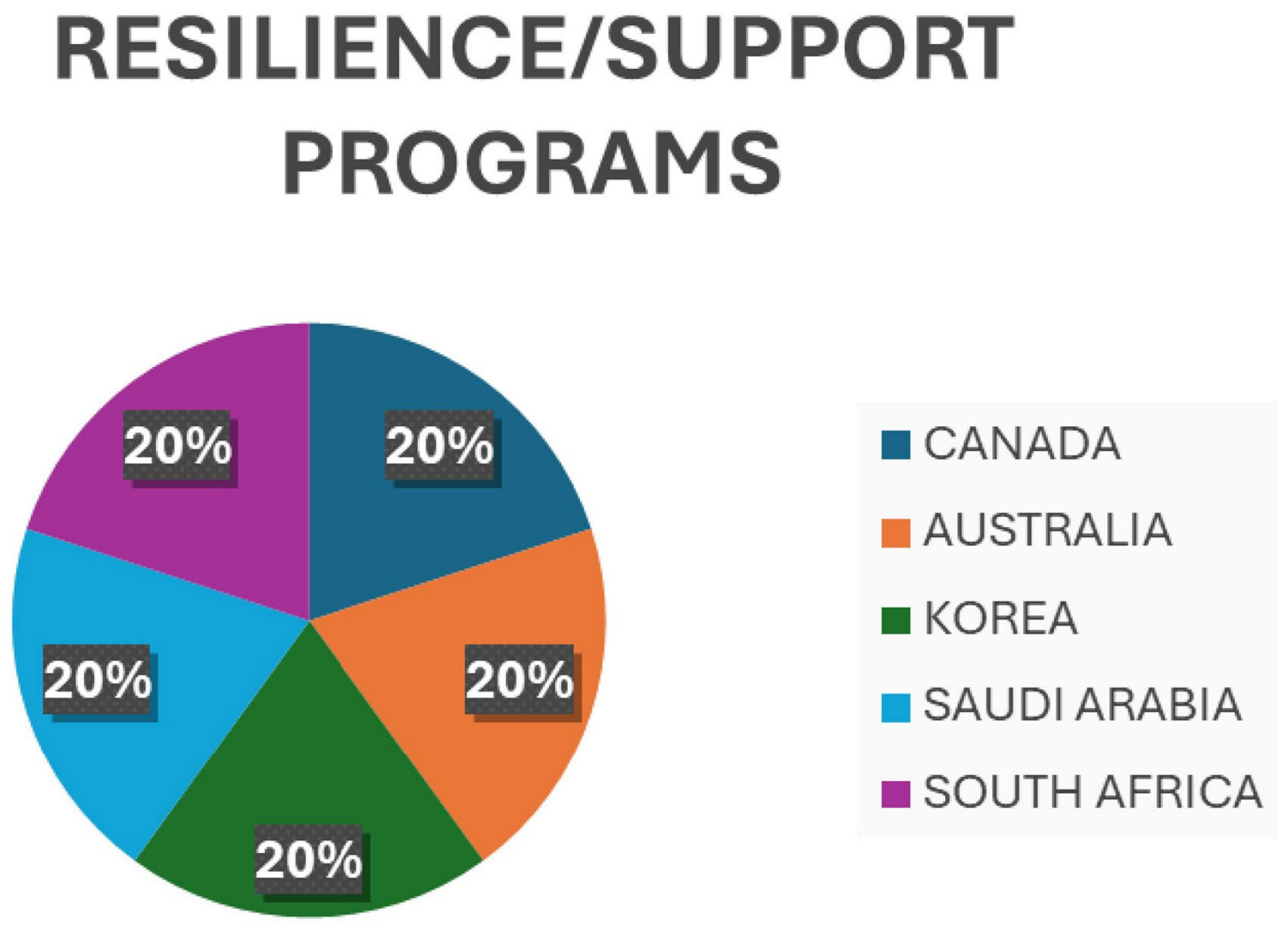
Percentage of resilience/support programs at individual level by region.

Evidence claimed by Lee and Chiyoung (44) supported reducing emotional exhaustion and depersonalization but did not support low personal accomplishment. Furthermore, MBI also effectively reduced the oncology nurses’ Compassion Fatigue. However, its effectiveness requires further research confirmation (45, 46). *Art Therapy*: One of the included reviews revealed significant reductions in anxiety, depression, and perceived stress levels among clinical nurses (47). *Music-based interventions*: Another review suggested that music interventions may decrease stress parameters even under critical stressful pressure (19). *Interventions based on cognitive behavioral therapy (CBT)* for PTSD, anxiety, and depression were found to lead to reliable changes in PTSD and anxiety symptoms (48). Some interventions were also found based on resilience programs, mobile applications, and nurse-led interventions that showed some effects on secondary traumatic stress, burnout, and compassion satisfaction (45). Figures 2–5 describe individual interventions by region.

##### 3.5.2.2 Organizational-level interventions

The review identified a glaring lack of organizational-level intervention in coping with emotional distress and improving work-related outcomes. However, only one of the reviews concluded that increasing salaries, nurse remuneration or benefits, improving professional skills, empowerment, strengthening mutual support among peers, and engaging in team-building activities can help reduce work alienation, considering these factors as a protective factor (49).

#### 3.5.3 Policy-level interventions

No specific policy interventions were found, but some authors pointed out some recommendations based on their findings. Recommendations included highlighting the need for gender-sensitive policies and programs when addressing the diverse effects of the pandemic on different employment outcomes, such as recent precedents on healthcare pressure and the impact on the mental health and well-being of health and care workforce, they also show that further studies are required to establish successful methods to promote gender equality in the labour market and to point out the underlying causes of the gender differences detected through the analysis (50).

## 4 Discussion

The Our Duty of Care report highlights the significant mental health challenges confronting the health and care workforce, exacerbated by the COVID-19 pandemic (15, 51). Contributing factors include excessive workloads, inadequate pay, high patient- to-nurse ratios, early burnout, and increasing intention to leave the profession. Additionally, societal shifts such as ageing populations and the rise of chronic illnesses have amplified the demand for nurses, particularly in community and long-term care settings (52–54).

Our review highlighted the need for systemic changes to safeguard HCWs’ mental health and well-being in the post-COVID-19 era. Despite extensive evidence of the mental health burden and effective interventions, there is a notable lack of systemic implementation and organizational accountability. A significant shift is necessary to address ongoing issues like anxiety, depression, burnout, stress, and PTSD among HCWs (22, 36, 50, 55–58). However, depression prevalence data must be cautiously interpreted due to inconsistencies in definitions and measurement methods (23).

Galanis et al. (33) recently found that high levels of compassion satisfaction among nurses reduced workplace bullying’s negative effects, lowering the risk of compassion fatigue, burnout, stress, and depression. This finding should inform future organizational and individual interventions aimed at improving HCWs’ mental health and well-being.

### 4.1 Shifting the burden: from individual resilience to organizational resilient culture

The COVID-19 pandemic exposed the limitations of focusing solely on individual resilience, highlighting the need to integrate resilience into organizational culture. The Our Duty of Care report (15) highlighted the failure to adequately support HCWs, emphasizing the importance of sustainable interventions. Placing the burden of resilience solely on individuals can harm their mental health, especially when systemic factors are ignored. Evidence suggests that healthcare organizations must provide supportive environments during crises, which enhances both healthcare systems and patient care (33, 39, 59). Therefore, a fundamental shift in organizational culture is needed to foster a resilient healthcare system.

Resilience operates across multiple levels: individual, organizational, and systemic. In times of crisis, resilience should be initiated at the organizational level, as healthcare organizations play a pivotal role in supporting health and care workforce (60, 61). By integrating structural, non-structural, and functional elements of resilience, organizations can effectively support HCWs during disruptions, alleviating individual stress and enhancing crisis management at the system-wide level.

The focus on resilience has evolved beyond individuals to exploring how organizations can foster resilience within their systems. Recent efforts aim to understand the human elements of resilient systems, including how resilience affects health and care workers, patients, and families. This shift emphasizes informal networks, communication, and the importance of engaging people in maintaining system safety. Additionally, there is a need for further research on everyday clinical work and developing robust research methods, such as simulations, to study resilience in healthcare systems (62).

A resilient healthcare system is characterized by robust leadership, adequate resources, and effective organization. Resilience requires a motivated workforce, strong partnerships, and reliable information systems, alongside financial reserves. Effective leadership is essential to mobilize resources, adapt service delivery, and manage crises. Policymakers must regularly assess their healthcare systems to identify vulnerabilities and prepare for potential risks. Targeting weak areas enhances both resilience and overall system performance (63).

Lessons learned from recent shocks offer valuable insights into building resilient healthcare systems. While effective crisis responses are essential, a comprehensive understanding of resilience necessitates an examination of its broader dimensions, particularly the integration of healthcare systems with external frameworks, such as ecological and socio-political structures. Well-integrated systems demonstrate greater resilience in withstanding shocks. Furthermore, crises often exacerbate existing inequalities, intensifying disparities in health outcomes and access to care. Addressing these inequities is a critical component of resilience-building policies (64).

The transition from merely conceptualizing resilience to actively operationalizing it within healthcare systems is imperative. Strengthening resilience requires the creation of enabling environments through the development of policies, operational frameworks, and evaluation mechanisms. Additionally, integrating health and care workers into governance structures and enhancing their capacities are vital steps in constructing a more resilient healthcare system (65).

### 4.2 Organizational interventions: a critical need

After analyzing the 50 included articles, no organizational-level interventions aimed at addressing emotional distress and improving work-related outcomes were observed. Review findings also highlighted the lack of training for managers in specific mental health conditions of HCWs. The findings emphasize a considerable need for equipping leaders with skills to help create a supportive environment for the health and care workforce and address the mental health crisis in the workplace. Findings also indicate that organizations with supportive environments facilitate more effective crisis management, enhance work satisfaction, and highlight the significance of fostering resilience in organizations (39, 59). This further emphasizes the importance of providing leaders with the necessary resources to establish such a setting and proactively tackle mental health issues.

Although Cognitive Behavioral Therapy (CBT) and mindfulness interventions have been shown to be effective at the individual level, their integration into organizational-level interventions remains limited (29, 41–43, 48, 66). There is a need for healthcare systems to adopt evidence-based organizational interventions that promote psychological well-being and job satisfaction on a broader scale (38, 39, 59, 67, 68).

Therefore, considering all possible scenarios and stakeholders designing complex interventions attempts to go beyond asking whether an intervention works in terms of achieving the intended outcome, and address a broader range of questions (e.g., identifying the impact on HCW, patient outcomes, economic impact on the organization and the healthcare system). Furthermore, designing complex interventions for system change could use the evidence generated to support real-world decision-making. A complex intervention is one that is designed taking into account other components than the intervention itself, allowing its implementation to vary in different contexts, while maintaining the integrity of the core components of the intervention taking into account the context, key uncertainties, stakeholders, economic considerations and continuous improvement of the intervention based on the aforementioned components, with the aim of refining and adapting it to the context and need (69).

### 4.3 Assessment tools: a gap in systemic evaluation

Although the mental health burden among HCW has been acknowledged (20, 57, 58), there is a noticeable lack of comprehensive assessment tools designed to evaluate and improve organizational interventions. Existing tools primarily assess individual mental health factors without offering a comprehensive framework for identifying and addressing issues at an early stage. Findings signify the need for developing comprehensive guidelines for assessing organizational and individual interventions (38, 68, 70–74), both universal and specific, to evaluate the implementation and effectiveness of interventions in healthcare organizations to enhance HCWs’ mental health and well-being.

### 4.4 Strengths and limitations of this rapid review

Although we performed a rigorous rapid review following PRISMA guidelines, our study had some limitations. First, we opted to use English as the language of inclusion to limit the number of articles we could gather, excluding other languages such as Spanish. Second, time constraints necessitated conducting the data analysis within a period of less than 2 months. Although most systematic reviews employed tools to assess the quality of the articles, we did not assess the thoroughness of these evaluations. All included articles were systematic reviews, with the majority having already evaluated the methodological quality of the studies within each review. No additional specific risk of bias assessment was performed on the included studies; however, we ensured that all studies had previously undergone a risk of bias evaluation.

Third, most of the reviewed studies focused on the mental health of nurses and physicians. This emphasis should be considered when applying the findings to other healthcare and care workers. Although this rapid review included studies on other healthcare professionals, such as dentists, psychologists, and physiotherapists, these groups were underrepresented or, in some cases, not studied at all. Fourth, this rapid review excluded qualitative studies, as quantitative research typically involves larger sample sizes and employs standardized measures, making its findings more generalizable. Nonetheless, qualitative research remains essential for capturing the complexities of mental health and well-being, offering valuable insights into the personal, emotional, and cultural factors that may not be adequately addressed through quantitative methods. While quantitative approaches are often prioritized when seeking large-scale, standardized evidence, both methodologies are complementary and contribute to a more comprehensive understanding of the topic. Future reviews would benefit from a mixed-methods approach, incorporating both qualitative and quantitative studies to provide a more comprehensive understanding of the topic.

## 5 Conclusion

### 5.1 Towards health system resilience

The findings of this review indicate that, although there has been no significant change in the prevalence of depression and anxiety among health and care workers since 2022, these workers continue to exhibit levels of anxiety and depression. These levels are clearly representative in specific groups of workers and units. This suggests that these mental health issues and burnout are not exclusive to pandemic situations but may be associated with work-related factors, as well as personal and family factors of each worker. Therefore, the findings of this review underscore the significant mental health challenges faced by health and care workers, which are exacerbated by systemic issues such as inadequate working conditions, staff shortages, and evolving societal demands.

Based on the analysis of the interventions offered to health and care workers (HCWs), it was observed that individual interventions are the most commonly provided. These interventions have shown some effectiveness in reducing stress, improving well-being, and developing coping strategies. Among the most notable individual interventions are mindfulness and cognitive-behavioral therapy. However, it is crucial to address the underlying causes of burnout, stress, and anxiety, as this requires broader structural changes within healthcare organizations.

It is evident that relying solely on individual resilience is insufficient and unsustainable. Instead, resilience must be embedded within healthcare systems, with organizations assuming a central role in fostering supportive environments. This necessitates the integration of structural, non-structural, and functional elements of resilience into organizational frameworks. By adopting these strategies, healthcare organizations can not only enhance support for their health and care workforce but also improve overall patient care and system performance, particularly in times of crisis.

The broader implications of these findings highlight the significant, far-reaching consequences of poor mental health among HCWs. Beyond the direct impact on individuals, these challenges affect patient outcomes, healthcare quality, and the efficiency of healthcare systems. To mitigate these issues effectively, a shift towards evidence-based, systemic interventions is required, moving beyond the limited scope of individual-level solutions.

Finally, the findings highlighted the lack of specific policies, with some authors recommending the establishment of policies and programs that consider all stakeholders. Therefore, it is imperative to develop comprehensive guidelines for healthcare organizations to safeguard the mental health and well-being of health and care workers. These guidelines should be aligned with international standards, such as those set by the WHO, and should emphasize systemic accountability, organizational interventions, and continuous evaluation of their effectiveness. Implementing these guidelines is essential to foster a supportive and healthy work environment, more resilient healthcare systems, enhanced workforce retention, and improved patient care outcomes. Safeguarding the mental health of health and care workers is not only an ethical responsibility but also a critical foundation for building resilient and sustainable healthcare systems.

## 6 Recommendations

Evidence indicates the necessity of proposing and incorporating policies to integrate mental health support into workplace practices. Consequently, organizational strategies that foster resilience, such as adjusting workloads and enhancing peer support, are emphasized.

Incorporation of mental health support in workplace policies

- Develop employee-sensitive mental health policies specific to the needs of health and care workers (HCWs).
- Institutionalise accessible psychological support services at all organisational levels, such as well-being centres and counselling programs.
- Implement Employee Assistance Programs (EAPs) to promote early identification and intervention for mental health concerns among HCWs.
- Implement programs that build awareness and develop psychological safety among health and care workers through ruptured programs.

Resilience-based organisational practices

- Modify workload demands and task distributions to sustainable levels to prevent burnout.
- Strengthen peer support systems by creating opportunities for HCWs to share experiences and coping strategies.
- Adopt multi-component interventions, such as training on personal protective equipment use, emotional first aid desks, and structured resilience-building programs.

Leadership and managerial support

- Train leaders and managers to identify and respond to mental health challenges among their staff effectively.
- Integrate mental health into organisational crisis management frameworks with an emphasis on the role of leaders in supporting HCWs during disruptions.
- Allocate resources for mental health support and resilience enhancement within healthcare settings.
- Establish frameworks for periodic evaluations of healthcare facilities to detect vulnerability areas and engage in preventive practices.

To policymakers

- Design policies that introduce mental health support in organisational settings. This aims to bring about systemic/organisational responsibility.
- Invest in comprehensive interventions targeting HCW mental health, including resilience-building initiatives and welfare programs.

For healthcare organizations

- Invest in managerial training to create supportive work environments that enable early identification of signs of mental distress among staff.
- Focus organisational interventions on reducing burnout through workload adjustments and improvement in peer support systems.
- Regularly review the effectiveness of implemented interventions with validated assessment tools and make improvements accordingly.

For researchers

- Conduct longitudinal studies to determine the sustained effects of organisational and individual-level interventions on HCW mental health.
- Develop and validate assessment tools tailored to organisational resilience and mental health interventions.
- Examine the interaction between sociodemographic factors and intervention outcomes to inform context-specific strategies.
- These recommendations are intended to fill systemic gaps pointed out in the manuscript to create a resilient and supportive healthcare environment that prioritises the well-being of its workforce.
