# Supplementary material for: Strategies to support the mental health and well-being of health and care workforce: a rapid review of reviews
Source: Front Med (Lausanne). 2025 Mar 19;12:1530287. doi: 10.3389/fmed.2025.1530287 (PMC11961965; doi:10.3389/fmed.2025.1530287)
Supplement: Supplementary file 1 [file Table_1.docx]

| **Year** | **Author** | **Title** | **Study Design** | **Country of Publication** | **Sample Size** | **Types of healthcare workers** | **Assessment tool used to evaluate MH and WB outcomes** | **Aims** |
| --- | --- | --- | --- | --- | --- | --- | --- | --- |
| 2022 | Thielmann, Beatrice; Schnell, Julia; Böckelmann, Irina; Schumann, Heiko | Analysis of Work Related Factors, Behavior, Well-Being Outcome, and Job Satisfaction of Workers of Emergency Medical Service: A Systematic Review | Systematic Review | Germany | 31.668 | Paramedics, Emergency medical services professionals, emergency medical technicians, drivers, emergency physicians | MBI, HSE, NEO-FFI, PSQI, STAI, BDI, Davidson Trauma Scale, Emotional Empathy task, PTGI, IES-R, R-COPE, AUDIT, BCI, Job Stress Survey, CMC, GHQ-28, IES, HS, PMI, AWSQ, PDS, HADS, CAQ, PDL-L, EDI, SOP-2, OSSS, MOB-L, CAR, HR, TICS, PSS, SF-PCL-5, Checklist for occupational stressors, CSD, social conflict, 3-items of the Brief COPE, PEAT, ISSB, Perceived Prosocial Impact MHI-d, TAS-20, Job Satisfaction Questionnaire, M-PTSD, SOC, IES-15, PTSS-10, traumatic events, DTS, PSES, QEAW, CIS, CD-RISC, SFWL, GHQ-28, WHO-5, SWLS, WFC, Karolinska Sleep Questionnaire, SHC, Need for Recovery after Work Scale, BCI, RSES, JSS, MBI-HSS, Paykel’s Suicidal Feelings in the General Population questionnaire | The aim of this review was to analyze the literature on the associations between psychosocial or physical work factors on one hand and the well-being outcomes and job satisfaction on the other hand. |
| 2024 | Egbe, Azelle; El Boghdady, Michael | Anxiety and depression in surgeons: A systematic review | Systematic Review | UK | 11.399 | Surgeons | GAD-7, DASS-21, HADS, PHQ-9, PHQ-2, GHQ-12 | To review the prevalence of anxiety and depression among surgeons over the past 10 years, to identify factors associated with anxiety and depression in surgeons and discuss potential methods to address their impact. |
| 2022 | Nicolakakis, Nektaria; Lafantaisie, Maude; Letellier, Marie-Claude; Biron, Caroline; Vézina, Michel; Jauvin, Nathalie; Vivion, Maryline; Pelletier, Mariève | Are Organizational Interventions Effective in Protecting Healthcare Worker Mental Health during Epidemics/Pandemics? A Systematic Literature Review | Systematic Review | Canada | Not mentioned | Nurses and doctors | PSQI, GAD-7, PHQ-9, IES-R, MBI-EE, HSE-MSIT, NQoL-SAT-P | To evaluate the effectiveness of organizational and psychosocial work environment interventions to protect healthcare workers’ mental health in an epidemic/pandemic context. |
| 2024 | Galanis, Petros; Moisoglou, Ioannis; Katsiroumpa, Aglaia; Mastrogianni, Maria | Association between Workplace Bullying, Job Stress, and Professional Quality of Life in Nurses: A Systematic Review and Meta-Analysis | SR and Meta-Analysis | Greece | 3.730 | Nurses | Negative Acts Questionnaire-Revised, Likert, ProQOL. | to quantitatively summarize the data on the association between workplace bullying and job stress and the professional quality of life of nurses. |
| 2022 | Zheng, Qin; Yang, Kun; Zhao, Rui-Jie; Wang, Xue; Ping, Ping; Ou, Zheng-Hang; Su, Xiao-Peng; Zhang, Jing; Qu, Miao | Burnout among doctors in China through 2020: A systematic review and meta-analysis | SR and Meta-Analysis | China | 48.638 | Doctors | MBI-HSS, MBI-GS-A, MBI-GS-B, CMBI | To analyze surveys measuring the prevalence of burnout among Chinese doctors and reveal the overall prevalence, characteristics, timeline, and factors related to burnout. |
| 2022 | Hiver, Chloé; Villa, Antoine; Bellagamba, Gauthier; Lehucher-Michel, Marie-Pascale | Burnout prevalence among European physicians: a systematic review and meta-analysis | SR and Meta-Analysis | France | 27903 | Physicians | MBI General Survey (MBI-GS) and the MBI Human Services Survey (MBI-HSS)-No difference | To assess burnout prevalence rates among physicians practicing in Europe (regardless of their specialty) taking into account the main approaches used to defne burnout with the Maslach Burnout Inventory (MBI) tool. |
| 2022 | Moro, Juliana da Silva; Soares, Josiane Pezzini; Massignan, Carla; Oliveira, Luciana Butini; Ribeiro, Dayane Machado; Cardoso, Mariane; Canto, Graziela de Luca; Bolan, Michele | Burnout syndrome among dentists: a systematic review and meta-analysis | SR and Meta-Analysis | Brazil | 8823 | Dentists | MBI (on all 3 subscales) | To assess the prevalence of Burnout syndrome in dentists. |
| 2022 | Sauder, Matthew; Zagales, Israel; Zagales, Ruth; Das, Snigdha; Sen-Crowe, Brendon; Bilski, Tracy; Kornblith, Lucy; Elkbuli, Adel | Comprehensive Assessment of Burnout Among Surgical Trainees and Practicing Surgeons: A Systematic Review | Systematic Review | USA | N/S | Surgeons | MBI, WBI, Oldenburg Burnout Inventory, Professional Fulfillment Index, Mini-Z, Physician Well-Being Index, Shirom-Malamed Burnout Measure and others | To systematically characterize existing definitions of burnout, evaluate tools to measure burnout, and determine risk factors of surgical burnout. |
| 2022 | Aymerich, Claudia; Pedruzo, Borja; Pérez, Jose Luís; Laborda, Maria; Herrero, Jon; Blanco, Jorge; Mancebo, Gonzalo; Andrés, Lucía; Estévez, Olatz; Fern; ez, Maitane; Salazar de Pablo, Gonzalo; Catalan, Ana; González-Torres, Miguel Ángel | COVID-19 pandemic effects on health worker's mental health: Systematic review and meta-analysis | SR and Meta-Analysis | Spain | 271319 | Healthcare workers in general | PHQ9, SDS, HADS, BDI, CES-D, DASS-21, PHQ2/PHQ4, GAD-7, SAS, BAI, SATAI-S, CAS, SASRQ, PSS, IES-R, PCL-C, PC-PTSD, ISI, SQS, AIS, PSQI, MBI, CBI, Mini-z | To determine the updated, global frequency of these outcomes. |
| 2022 | Saade, Sabine; Parent-Lamarche, Annick; Bazarbachi, Zeina; Ezzeddine, Ruba; Ariss, Raya | Depressive symptoms in helping professions: a systematic review of prevalence rates and work-related risk factors | Systematic Review | Lebanon | 17437 | doctors, nurses, social workers, psychologists, psychiatrists, midwives, occupational therapists, speech pathologists, laboratory and X-ray technicians, community health workers, physical therapist, and eldercare workers | CES-D, HADS, BDI | To provide an overview of the prevalence rate of depression in a wide array of helping professions, and identify work organization conditions that seem to be associated with this depression risk. |
| 2024 | Musker, Mike; Othman, Shwikar | Effective interventions to reduce burnout in nurses: A meta-analysis | SR and Meta-Analysis | Australia | 3007 | Nurses and midwives | ProQOL, MBI | To examine the effectiveness of interventions to ameliorate burnout, secondary traumatic stress, and emotional exhaustion in nurses and midwives. |
| 2022 | Munhoz, Oclaris Lopes; Morais, Bruna Xavier; Santos, Wendel Mombaque Dos; Paula, Cristiane Cardoso de; Magnago, Tânia Solange Bosi de Souza | Effectiveness of auriculotherapy for anxiety, stress or burnout in health professionals: a network meta-analysis | SR and Meta-Analysis | Brazil | 860 | Healthcare workers in general | STAI, NRS, GAD, LSS, DASS-21, PQOL, | To analyze the effectiveness of auriculotherapy, when compared to the control group, placebo or usual treatment for anxiety, stress or burnout in health professionals. |
| 2022 | Ottisova, Livia; Gillard, Julia A.; Wood, Maximillian; Langford, Sarah; John-Baptiste Bastien, Rayanne; Madinah Haris, Aishah; Wild, Jennifer; Bloomfield, Michael A. P.; Robertson, Mary | Effectiveness of psychosocial interventions in mitigating adverse mental health outcomes among disaster-exposed health care workers: A systematic review | Systematic Review | UK | Not defined | Healthcare workers in general | CES-D, HADS, BDI | To summarize the evidence for the interventions in preventing or reducing adverse mental health outcomes in health care staff, with particular emphasis on PTSD, anxiety, depression, and sleep difficulties; appraise the effectiveness of the interventions on psychological outcomes by calculating indices of reliable change for primary outcome measures; and provide clinical recommendations. |
| 2023 | Wang Q; Wang F; Zhang S; Liu C; Feng Y; Chen J | Effects of a mindfulness-based interventions on stress, burnout in nurses: a systematic review and meta-analysis. | SR and Meta-Analysis | China | 1165 | Nurses | CES-D, HADS, BDI, MBI | To systematically evaluate the impact of mindfulness training on nurse’s performance and increase the certainty of existing evidence. |
| 2024 | Zhou, X.-Q.; Han, Y.-F.; Xu, M.-X. | Effects of different intervention methods on psychological anxiety, stress, and fatigue among healthcare workers during COVID-19 pandemic: a systematic review and meta-analysis | SR and Meta-Analysis | China | 1466 | Healthcare workers in general | GAD-7, PSS-4, MBI | To explore the treatment effects of various intervention methods on the stress, anxiety, and fatigue of medical workers during the 2019 Coronavirus Disease (COVID-19) pandemic. |
| 2022 | Kang MJ; Myung SK | Effects of Mindfulness-Based Interventions on Mental Health in Nurses: A Meta-Analysis of Randomized Controlled Trials. | SR and Meta-Analysis | Korea | 572 | Nurses | SAS, SDS, GAD-7, POMS-TA, STAI, SCL-90, PHQ-9, PSS, Irritation scale, MBI, PANAS, CD-RISC, WHO-5, WHOQOL-BREF, MAAS, MMSS, FMI, TAA, WAI, CES, Neal, Griffin and Hart's measure workplace | To evaluated the psychological effects of mindfulness-based interventions (MBIs) on mental health in nurses. |
| 2023 | Karo M; Simorangkir L; Daryanti Saragih I; Suarilah I; Tzeng HM | Effects of mindfulness-based interventions on reducing psychological distress among nurses: A systematic review and meta-analysis of randomized controlled trials. | SR and Meta-Analysis | Indonesia | 1372 | Nurses | PSS, GAD-7, STSS, PROMIS, PH1-9, HADS, C-STAI, BAI | To examine the effects of mindfulness-based interventions on reducing stress, anxiety, and depression among nurses. |
| 2023 | Yang, Jia-Ming; Ye, Hua; Long, Yi; Zhu, Qiang; Huang, Hui; Zhong, Yan-Biao; Luo, Yun; Yang, Lei; Wang, Mao-Yuan | Effects of Web-Based Mindfulness-Based Interventions on Anxiety, Depression, and Stress Among Frontline Health Care Workers During the COVID-19 Pandemic: Systematic Review and Meta-Analysis | SR and Meta-Analysis | China | 1311 | Healthcare workers in general | DASS-21, HADS, SHAI, PHQ-9, GAD-7, PSS, GAS, GDS, STAI-I, SAS, SDS | To explore whether web-based mindfulness-based interventions continue to have a positive impact on anxiety, depression, and stress among health care workers during the COVID-19 pandemic. |
| 2024 | Zhang, Na; Chen, Shuoxin; Li, Qing; He, Zhiqiang; Jiang, Wenhui | Efficacy of art therapy in enhancing mental health of clinical nurses: A meta‐analysis. | SR and Meta-Analysis | China | 1338 | Nurses | SAS, HADS, SCL-90, STAI, BAI, SDS, CPSS, CNSS, SCSQ | To conduct a systematic evaluation of the efficacy of art therapy on the mental health of clinical nurses |
| 2022 | Lim, Ji-Young; Kim, Geun-Myun; Kim, Eun-Joo | Factors Associated with Job Stress among Hospital Nurses: A Meta-Correlation Analysis | SR and Meta-Analysis | Korea | N/S | Nurses | MBI, PSS, HADS, GDS, GDS, STAI, WHO-5, WHOQOL-BREF | To investigate research trends concerning job stress among hospital nurses. |
| 2022 | Cheng, Crystal Kai Tian; Chua, Jie Hui; Cheng, Ling Jie; Ang, Wei How Darryl; Lau, Ying | Global prevalence of resilience in health care professionals: A systematic review, meta-analysis and meta-regression | SR and Meta-Analysis | Singapore | 17.073 | Healthcare workers in general | Block Ego-Resiliency Scale, BRCS, Brief Resilience Scale, Coonor Davidson Resilience Scale, 25-item Wagnild and Young Resilience Scale, 14-item Wagnild Resilience Scae, Thai-Resilience Questionnaire. Personal Views Survey and Dispositional Resilience Scale | To examine the prevalence estimate of low resilience among health care professionals and identify the factors affecting the prevalence. |
| 2023 | Papazian, Laurent; Hraiech, Sami; Loundou, Anderson; Herridge, Margaret S.; Boyer, Laurent | High-level burnout in physicians and nurses working in adult ICUs: a systematic review and meta-analysis | SR and Meta-Analysis | France | 20.723 | Physicians and nurses | MBI | To estimate the prevalence of high-level burnout in physicians and nurses working in adult ICUs |
| 2023 | Athe, Ramesh; Dwivedi, Rinshu; Singh, Kasha; Babusab Hulmani, Sabiha; Karadi, Nikhita; Boraiah, Chaithanya; Vasu, Sindhu | Impact of COVID-19 on the Mental Health of Healthcare Workers and Job Loss From a Gender Perspective in India: A Systematic Review and Meta-Analysis | SR and Meta-Analysis | India | >3420 | Healthcare workers in general | CES-D, HADS, BDI, MBI, GAD | To explore the gender perspective of mental health conditions among HCWs and job loss during the pandemic in India. |
| 2023 | Jiaru, Jiao; Yanxue, Zheng; Wennv, Hao | Incidence of stress among emergency nurses: A systematic review and meta-analysis | SR and Meta-Analysis | China | 4293 | Nurses | Stress questionnaire self-designed, Occupational stress source Questionnaire , Emergency nurses stress questionnaire, Chinese nurses work Stressors Scale, ENSS, HSS-35, Self-estimation scale Perceived stress scale, NSS, Chinese Perceived Stress Scales | To evaluate the stress level among emergency nurses systematically. |
| 2023 | Lee, Miran; Cha, Chiyoung | Interventions to reduce burnout among clinical nurses: systematic review and meta-analysis | SR and Meta-Analysis | Korea | 1935 | Nurses | MBI, ProQoL, burnout questionnaire and OLBI. | To evaluate burnout interventions for clinical nurses. |
| 2023 | Teng M; Yuan Z; He H; Wang J | Levels and influencing factors of mental workload among intensive care unit nurses: A systematic review and meta-analysis. | SR and Meta-Analysis | China | 4266 | Nurses | NASA-TLX questionnaire | To determine the levels and influencing factors of mental workload in intensive care unit nurses |
| 2024 | Huang, Jia; Huang, Zhu-Tang; Sun, Xin-Ce; Chen, Ting-Ting; Wu, Xiao-Tian | Mental health status and related factors influencing healthcare workers during the COVID-19 pandemic: A systematic review and meta-analysis | SR and Meta-Analysis | China | 341,014 | Healthcare workers in general | OLBI, MBI, SPFI, ProQoL, Mini-Z,STAI, GHQ-28, PROMIS, GAD-7, BAI, HADS, ISR, DASS, SAS, PHQ, CAS, SCL-90, HAM-D, IRS, BDI, SDS, CES-D, IES-R, PCL, DTS-8, ASDS | to systematically evaluate the mental health problems of healthcare workers worldwide during the pandemic and to determine the latest global frequency of COVID-19 associated mental health problems. |
| 2022 | Le Huu, Pierre; Bellagamba, Gauthier; Bouhadfane, Mouloud; Villa, Antoine; Lehucher, Marie-Pascale | Meta-analysis of effort-reward imbalance prevalence among physicians | SR and Meta-Analysis | France | 21,939 | Physicians | Effort–reward imbalance assessment tool | To estimate the efort–reward imbalance prevalence (ERI) among physicians. |
| 2022 | Chen, Yu; Wang, Jing; Geng, Yujie; Fang, Zhengmei; Zhu, Lijun; Chen, Yan; Yao, Yingshui | Meta-analysis of the prevalence of anxiety and depression among frontline healthcare workers during the COVID-19 pandemic | SR and Meta-Analysis | China | 18,382 | Healthcare workers in general | SAS, DASS-21, PHQ, GAD-7, HADS, The COVID-19 Anxiety Scale, BAI, BDI | To systematically review the prevalence of anxiety and depression among frontline healthcare workers during the coronavirus disease 2019 (COVID-19) pandemic. |
| 2023 | Colin, Catherine; Prince, Violaine; Bensoussan, Jean-Luc; Picot, Marie-Christine | Music therapy for health workers to reduce stress, mental workload and anxiety: a systematic review | Systematic Review | france | >29 | Healthcare workers in general | PSS-10, MASI-R, JSS or specific-study questionnaires), STAI-S, MBI, PILL questionnaire | To examine the efficacy of music interventions on stress parameters by selecting studies conducted in genuine care stress conditions. |
| 2022 | Khatatbeh, Haitham; Pakai, Annamária; Al-Dwaikat, Tariq; Onchonga, David; Amer, Faten; Prémusz, Viktória; Oláh, András | Nurses' burnout and quality of life: A systematic review and critical analysis of measures used | SR | Hungary | 9,859. | Nurses | MBI, CBI, Shirom–Melamed BO inventory. WHOQOL-BREF, SF-36 or SF-12, ProQOL. | To systemically review the relationship between nurses’ burnout and quality of life and to introduce practical recommendations to reduce nurses’ BO and improve their QOL. |
| 2023 | Xia M; Wang J; Bi D; He C; Mao H; Liu X; Feng L; Luo J; Huang F; Nordin R; Zakaria ZDH | Predictors of job burnout among Chinese nurses: a systematic review based on big data analysis. | Systematic Review | China | 1,057 | Nurses | MBI | To synthesize current research on predictors related to burnout affecting Chinese nurses. |
| 2024 | Zhang, Min; Bo, Mingyu; Wang, Huixin; Fan, Wenyi; Kong, Lingling; Zhou, Chunjie; Zhang, Zhenxing | Prevalence and influencing factors of post-traumatic stress disorder among Chinese healthcare workers during the COVID-19 epidemic: a systematic review and meta-analysis | SR and Meta-Analysis | China | 11,841 | Healthcare workers in general | IES-R; PCL-C; PCL-5 | To estimate the prevalence and influencing factors of post-traumatic stress disorder (PTSD) among Chinese healthcare workers during COVID-19 |
| 2023 | Harris, Natashia; Sheridan, Lorraine; Robertson, Noelle | Prevalence and Psychosocial Impacts of Stalking on Mental Health Professionals: A Systematic Review | SR | UK | 7,606 | Healthcare workers in general | BDI; STAI; Thee Rutgers-Penn Clinicians and Stalking questionnaire; | To assess prevalence of stalking, and associated impacts and methods of coping reported by mental health professionals, irrespective of perpetrator type. |
| 2022 | Algamdi, Maaidah | Prevalence of oncology nurses' compassion satisfaction and compassion fatigue: Systematic review and meta-analysis | SR and Meta-Analysis | Saudi Arabia | 2,509 | nurses | ProQOL scale | To systematically review and comprehensively analyse findings of studies reporting oncology nurses' compassion satisfaction, burnout and secondary traumatic stress, measured by the professional quality of life (ProQOL) scale |
| 2022 | Alberque, Bastien; Laporte, Catherine; Mondillon, Laurie; Baker, Julien S.; Mermillod, Martial; Brousse, George; Ugbolube, Ukadike Chris; Bagheri, Reza; Bouillon-Minois, Jean-Baptiste; Dutheil, Frédéric | Prevalence of Post-Traumatic Stress Disorder (PTSD) in Healthcare Workers following the First SARS-CoV Epidemic of 2003: A Systematic Review and Meta-Analysis | SR and Meta-Analysis | France | 4,842 | Healthcare workers in general | DTS-C:; IES/IES-R | To conduct a systematic review of the literature and meta-analysis on the prevalence of PTSD in HCWs. |
| 2023 | de Vargas D; Volpato RJ; Dos Santos LC; Pereira CF; de Oliveira SR; da Silva RR; Maciel MED; Fernandes IL; de Oliveira Santana K; Aguilar TF | Prevalence of psychological and mental health symptoms among nursing professionals during the COVID-19 pandemic in the Americas: Systematic review and meta-analysis. | SR and Meta-Analysis | Brazil | 52.270 | nurses | PHQ; GAD; MBI; DASS-21; TEPT; PCL; ISI | To assess the prevalence of mental health symptoms in nursing professionals during the COVID 19 pandemic on the American continent. |
| 2022 | You Q; Bai D; Wu C; Gao J; Hou C | Status of work alienation among nurses in China: A systematic review. | SR | China | 7,265 | nurses | The work alienation Chinese Scale | To systematically evaluate the status and distribution characteristics of work alienation among nurses in China. |
| 2022 | Bekelepi N; Martin P | Support interventions for nurses working in acute psychiatric units: A systematic review. | SR | South Africa | 296 | nurses | IWS; IES-R; POMS; DASS 21; SCL-90 scale; SDS; SAS; NSS; MBI | To examine effective stress reduction interventions for nurses and to identify key elements of these successful interventions. |
| 2022 | Claponea, Roxana Mihaela; Pop, Lavinia Maria; Iorga, Magdalena; Iurcov, Raluca | Symptoms of Burnout Syndrome among Physicians during the Outbreak of COVID-19 Pandemic-A Systematic Literature Review | SR | Romania | 3,071 | Physicians | MBI; BMS; Mini-Z Burnout; CBI; PFI; MBI-HSS; OLBI; CFST; FCV-19S; HADS | To assess the prevalence of burnout among physicians working in the healthcare system during the COVID-19 pandemic, and discovering the main factors associated with burnout syndrome among the population of physicians. |
| 2022 | Chen, Yuquan; You, Yanwei; Wang, Yue; Wang, Yudong; Dai, Tao | Systematic and meta-based evaluation on job satisfaction of village doctors: An urgent need for solution issue | SR | China | 23,595 | Doctors | UWES; The Chinese Physicians’ Job Satisfaction Questionnaire | To evaluate the village doctors’ job satisfaction status, to provide guidelines for the healthcare policies. |
| 2022 | Sulosaari V; Unal E; Cinar FI | The effectiveness of mindfulness-based interventions on the psychological well-being of nurses: A systematic review. | SR | Finland | 1,009 | nurses | DASS-21; JSS; MAAS; PSS; PANAS; CD-RISC; MMSS; ProQol5; GSES; SCS-SF; Brief Serenity Scale; Interpersonal Reactivity Index, Index of Work Satisfaction; MBI; GAD-7; WHO-HPQ, The EuroQol (EQ-5D). | To identify mindfulness-based interventions and outcome measures and to evaluate the effect on the psychological well-being of nurses. |
| 2022 | Yoo, In Gyu | The effects of the type of delivery of cognitive-behavioral therapy for healthcare workers: A systematic review | SR | Korea | 3,701 | nurses | PSS, Korean Occupational Stress Scale; NSS; Hospital Service Stress; MBI; DASS‐21; PSQI; ESS;GSSS; the Relationship Change Scale‐Korean version, Negative Acts Questionnaire‐Korean version, Turnover intention tool; the Sense of Coherence, Profile of Mood States; Brief Symptom Inventory‐18, CESDS; Short Warwick‐Edinburgh Mental Well‐being Scale; BDI‐2. | To assess the extent of knowledge of the effectiveness of cognitive‐behavioral therapy (CBT) for healthcare workers by type of delivery. |
| 2022 | Shen, Xin; Xu, Hongbin; Feng, Jing; Ye, Jun; Lu, Zuxun; Gan, Yong | The global prevalence of burnout among general practitioners: a systematic review and meta-analysis | SR and Meta-Analysis | China | 7,595 | Doctors | MBI scale | To systematically measured the global prevalence of burnout among GPs. |
| 2022 | Johns, G.; Samuel, V.; Freemantle, L.; Lewis, J.; Waddington, L. | The global prevalence of depression and anxiety among doctors during the covid-19 pandemic: Systematic review and meta-analysis | SR and Meta-Analysis | UK | 33,281 | Doctors | GAD-7; PHQ-9; HADS; DASS-21; PHQ-2, GAD-2; BAI; PROMIS | To analyse the evidence emerging from the first year of the COVID-19 pandemic |
| 2024 | Wang, Jinfeng; Luo, Zhipeng; Liao, Xinqi; Zeng, Yanli; Zhou, Jing; Liu, Minyan; Yao, Yue; Tian, Jie; Luo, Wanting | The levels and related factors of posttraumatic growth among nurses: A systematic review and meta-analysis | SR and Meta-Analysis | China | 35,621 | Nurses | PTGI/PTGI-SF: Post traumatic Growth Inventory Short Form | To explore the levels and related factors of PTG among nurses. |
| 2023 | Bai, Xiaoyin; Wan, Ziqi; Tang, Jieying; Zhang, Dingding; Shen, Kaini; Wu, Xia; Qiao, Lin; Zhou, Yangzhong; Wang, Yaqi; Cheng, Wei; Jiang, Wei; Wang, Luo; Tian, Xinlun | The prevalence of burnout among pulmonologists or respiratory therapists pre- and post-COVID-19: a systematic review and meta-analysis | SR and Meta-Analysis | China | 3610 | Doctors | MBI; ProQOL: professional quality of life scale; SPFI: Stanford Professional Fulfilment Index; WBI | To compare the differences in burnout among pulmonologists or respiratory therapists pre- and post-COVID 19 by doing a systematic review with meta-analysis. |
| 2023 | Long, Huiqing; Li, Qingshu; Zhong, Xiaogang; Yang, Lu; Liu, Yiyun; Pu, Juncai; Yan, Li; Ji, Ping; Jin, Xin | The prevalence of professional burnout among dentists: a systematic review and meta-analysis | SR and Meta-Analysis | China | 6,039 | Dentist | MBI | To investigate the prevalence of professional burnout among dentists. |
| 2023 | Ma, Yao; Xie, Tian; Zhang, Jian; Yang, Hui | The prevalence, related factors and interventions of oncology nurses' burnout in different continents: A systematic review and meta-analysis | SR and Meta-Analysis | China | 5.304 | Nurses | MBI | To investigate the prevalence, influencing factors and interventions of oncology nurses' burnout among different continents. |
| 2022 | Xiong, Nana; Fritzsche, Kurt; Pan, Yiqi; Löhlein, Johanna; Leonhart, Rainer | The psychological impact of COVID-19 on Chinese healthcare workers: a systematic review and meta-analysis | SR and Meta-Analysis | China | 65,706 | Healthcare workers in general | PCL-C; PTSD-SS;PCL-5; SASRQ;SAS; GAD-7; SCL-90; HAM-A; BAI; HADS; PHQ-9; SCL-90; CES-D; Beck Depression Inventory (BDI-II); PSQI; ISI; MBI | To investigate five dimensions of the psychological impact (post-traumatic stress symptoms (PTSS), anxiety, depression, sleep disturbance or profession-related burnout) of COVID-19 on healthcare workers (HCW) in China |
| PTSD: Posttraumatic stress disorder; PSQI: Pittsburgh Sleep Quality Index; STAI: State-trait anxiety inventory; BDI:Beck depression inventory; PTGI: Posttraumatic Growth Inventory; IES-R: Impact of Events Scale-Revised; R-Cope: R-COPE Inventory; NEO-FFI: revised Costa and McCrae; BCI: Basic Character Inventory; GHQ: General Health Questionnaire; IES/IES-R/IES-15: The Impact of Event Scale/-Revised; MBI/MBI-HSS: Maslach Burnout Inventory/MBI-Human Services Survey; HSE: Health and Safety Executive Job Stress Questionnaire; AWSQ: Ambulance Work Stressors Questionnaire; HADS: Hospital anxiety and depression scale; PCL-C: Posttraumatic stress disorder checklist-civilian version; SF-36: short form Health survey-36; SOP-2: Optimism-Pessimism-2 Scale; OSSS: Oslo Social Support Scale; PHQ-4: Patient Health Questionnaire 4; CAR: Cortisol awakening response; PSS: Perceived stress scale; CBI: Copenhagen Burnout Inventory; CSD: Consensus Sleep diary; DTS: Davidson Trauma scale for PTSD; AUDIT: Alcohol Use Disorders Identification Text; QEAW: Questionnaire on the Experience and Assessment of Work; CIS: Checklist of Individual Strength.; GAD-7: Generalised Anxiety Disorder Assessment-7; DASS-21: Depression, Anxiety and Stress Scale-21; NQoL-SAT-P: Nurses Quality of Life Scale−Satisfaction Profile; SAS: Zung Self-Rating Anxiety Scale; BAI: Beck Anxiety Inventory; CAS: Coronavirus Anxiety Scale; SARSQ: Stanford Acute Stress Reaction Questionnaire; ISI: Insomnia Severity Index; SQS: Sleep Quality Scale; Mini-Z: Mini-Z Burnout Survey; PANAS: Positive and Negative Affect Schedule; CD-RISC: Connor–Davidson Resilience Scale; MMSS: The McCloskey/Mueller Satisfaction Scale; MMSS: The McCloskey/Mueller Satisfaction Scale; SAS: The self -rating anxiety scale; WHOQOL-BREF: World Health Organization Quality of Life-BREF; WAI: Workability Index Scale; ENSS: Expanded nursing work stress scale; HSS-35: The hospital stress scale; OLBI: Oldenburg Burnout Inventory; PROMIS: National Institution of Mental Health; HAM-D: Hamilton Depression Scale; ISR: The self-report questionnaire; ASDS: Acute Stress Disorder Scale; DTS-8: The Davidson Trauma Scale; IES-R: The 22-item Impact of Event Scale-Revised; MASI-R: Maugeri Stress Index-Revised; IWS: Lee’s Index of Work Satisfaction; POMS: Profile Mood State;BMS: The 10-item Burnout Measure Short Version; CFDT: The Compassion Fatigue and Satisfaction Self-Test; FCV-19S: The Fear of COVID-19 scale; UWES: Utrecht Work Engagement Scale; PANAS: Positive and Negative Affect Schedule; GSES: The General Self-Efficacy Scale | | | | | | | | |
