# Supplementary material for: Strategies to support the mental health and well-being of health and care workforce: a rapid review of reviews
Source: Front Med (Lausanne). 2025 Mar 19;12:1530287. doi: 10.3389/fmed.2025.1530287 (PMC11961965; doi:10.3389/fmed.2025.1530287)
Supplement: Supplementary file 2 [file Table_2.docx]

| **Reference** | **Sample** | **Mental Health Burden** | | | | | **Well-being** | | | | | | **Work environment** | | **Type of intervention** | | **Policy-level Interventions** |
| --- | --- | --- | --- | --- | --- | --- | --- | --- | --- | --- | --- | --- | --- | --- | --- | --- | --- |
|  |  | **Burnout** | **Anxiety** | **Depression** | **PTSD** | **Others** | **Job satisfaction** | **stress/Job stress** | **Sleep disturbance** | **QoL** | **General health** | **Others** | **Workplace bullying** | **Others** | **Universal organizational intervention** | **Individual/Psychological intervention** |  |
| Thielmann et al., 2022. Germany | Paramedics, Emergency medical services professionals, emergency medical technicians, drivers, emergency physicians | the prevalence of burnout was 8%. The predictors of burnout were time pressure (adjusted odds ratio, AOR: 4.4), working 12 or more shifts ≥24h in the past 30 days, working 12 or more night shifts in the past 30 days. | The level of anxiety symptoms was lower than for the general population, especially for men and women | The level of depression symptoms was also lower than for the general population (for men). | The prevalence rate of PTSD was 5.6%; for partial PTSD, the rate was 15%. EMS with PTSD symptoms had more frequent self-reported emotional problems, more medical visits, poorer QoL, and poorer general health. | 18.8% of men and 10.7% of women used alcohol to cope. 22.8% reported a lifetime suicidal ideation, with 10.4% of those reporting serious suicidal ideation and 3.1% reporting having previously attempted suicide. No gender difference was found, but there was a higher prevalence of women who ‘wished they were dead’ | 60% indicated job satisfaction at all, and 22% indicated a high level of job satisfacti | Not mentioned | Not mentioned | Not mentioned | Various physical symptoms were present under physical stress conditions: 66.6% of the respondents reported physical stress (42.6% with symptoms/pain) in the lower back, 36.7% (17.1% with symptoms/pain) in the neck, 27.9% (14.1% with symptoms/pain) in the knees, 24.3% (12.3% with symptoms/pain) in the upper back, and 33.5% (16.4% with symptoms/pain) in the shoulders | Job resources such as supervisor respect or management support and schedule control helped reduce burnout. | 61.7% reported that violence occurred during the night shift. Only 10% received psychological or legal support. A total of 63.3% found a lack of support from administrative directors in cases of exposure to violence, which led to the experience of fear of violence. | That the workplace does not include a place to exercise, relax or eat | Not mentioned | Not mentioned | Not mentioned |
| Egbe et al., 2024 | Surgeons | There was a substantial incidence of burnout associated with other mental health conditions. | The median percentage of anxiety was 20 with a range of 54.6%. | the median of depression percentage was 24% with a range of 59%. | Not mentioned | One study found the rate of suicidal thoughts to be 1.5 to 3 times higher than the national average, but only 26% of those with suicidal thoughts had sought help, and 60% of those with suicidal ideation were hesitant to seek help due to concerns about their career. One study found a high level of mental health disorders including drugs abuse. | Not mentioned | Not mentioned | Not mentioned | Not mentioned | Not mentioned | Not mentioned | Not mentioned | Not mentioned | Not mentioned | Not mentioned | Not mentioned |
| Nicolakakis et al., 2022 | Nurses and doctors | Not mentioned | Recognition measures are associated with 24% ↓ likelihood of anxiety (GAD-7 ≥ 8) compared to not having received recognition measures: AOR (95% CI): 0.76 (0.60–0.97); p = 0.03 | Recognition measures are associated with 31% ↓ likelihood of depression (PHQ-9 ≥ 10) compared to not having received recognition measures: AOR (95% CI): 0.69 (0.52–0.90); p = 0.007 | Not mentioned | Not mentioned | ↑ work engagement of 0.19 points on dedication subscale of UWES-9 ranging from 0 to 6 points: mean score (SD) is 5.02 (1.38) for center users and 4.83 (1.15) for non-users; p = 0.08 | Recognition measures are associated with 24% ↓ likelihood of acute stress in the past 7 days caused by a traumatic event, COVID-19 being the specific event (IES-R > 33), compared to not having received recognition measures: AOR (95% CI): 0.76 (0.60–0.97); p = 0.024 | Not mentioned | Not mentioned | 6= % presenteeism past 12 months among center users vs. non-users: no, never: 16.31 vs. 14.97 yes, once: 17.05 vs. 12.76 yes, 2 to 5 times: 16.92 vs. 12.64 yes, >5 times: 4.53 vs. 4.41 p = 0.28 | ↑ mental wellbeing of 1.93 points on WEMWBS scale that ranges from 14 to 70 points: mean WEMWBS score (SD) is 47.04 (9.49) for center users and 45.11 (9.35) for non-users; p = 0.02 | ↑ quality of relationships at work (harassment, tension, bullying) post-program: mean HSE-MSIT subscale score (SD) on scale of 1 to 5: T0: 2.23 (0.88) vs. T1: 2.04 (0.68); p = 0.001 | ↑ quality of the psychosocial work environment post-program: mean HSE-MSIT score (SD) on scale of 1 to 5: T0: 2.46 (0.40) vs. T1: 2.32 (0.50); p < 0.001. No differences on work demands (workload, time pressure) post-program: mean HSE-MSIT subscale score (SD) on scale of 1 to 5: T0: 2.81 (0.48) vs. T1: 2.79 (0.58); p = 0.601 | Not mentioned | Simulation-based teamwork training programm and Wellbeing centers supported. Multi-component SARS prevention program. “R2 for Leaders” resilience training program. Multi-component COVID-19  prevention program:  reorganized wards | Not mentioned |
| Galanis et al, 2024 | Nurses | The meta-regression analysis showed that the association between workplace bullying and job burnout was stronger in studies with a higher percentage of females (coefficient beta = 0.01, 95% CI = 0.006 to 0.019, p = 0.001). | Not mentioned directly but the correlation of the variables is discussed | Not mentioned directly but the correlation of the variables is discussed | Not mentioned | Not mentioned | Not mentioned directly but the correlation of the variables is discussed | Regarding secondary traumatic stress, the correlation coefficients ranged from 0.02 to 0.53 and were statistically significant in two out of three studies. The pooled correlation coefficient was 0.36 | Not mentioned directly but the correlation of the variables is discussed | Not mentioned directly but the correlation of the variables is discussed | Not mentioned directly but the correlation of the variables is discussed | Not mentioned | correlation coefficient between workplace bullying and job stress. The correlation coefficients ranged from 0.31 to 0.37 and were statistically significant (p < 0.001 in all cases) | Not mentioned | Not mentioned | Not mentioned | Not mentioned |
| Zheng et al., 2022 | Doctors | The overall prevalence of burnout was 75.48% (95% CI, 69.20 to 81.26; I2 1⁄4 99.23%, P < 0.001), and high burnout was 9.37% (95% CI, 4.91 to 15.05, I2 1⁄4 98.88%, P < 0.001). | Not mentioned directly | Not mentioned directly | Not mentioned directly | Not mentioned | Not mentioned directly | Not mentioned directly | Not mentioned directly | Not mentioned directly | Not mentioned | Not mentioned | Not mentioned | Not mentioned | Not mentioned | Not mentioned | Not mentioned |
| Hiver et al., 2022 | Physicians | Physicians’ burnout prevalence rates ranged from 2.5% to 72.0%. The pooled prevalence rate of burnout was estimated at 7.7% [5.3–10.4%] with the tridimensional defnition, 19.7% [13.5–26.3%] with the bidimensional defnition and 43.2% [29.0–57.6%] with the unidimensional defnition. | Not mentioned directly | Not mentioned directly | Not mentioned directly | Not mentioned | Not mentioned directly | Not mentioned directly | Not mentioned directly | Not mentioned directly | Not mentioned | Not mentioned | Not mentioned | Not mentioned | Not mentioned | Not mentioned | Not mentioned |
| Moro et al., 2022 | Dentists | The overall burnout syndrome prevalence in dentists found was 13% (95% confidence interval [CI]: 0.006-0.21) | Not mentioned directly | Not mentioned directly | Not mentioned directly | Not mentioned | Not mentioned directly | Not mentioned directly | Not mentioned directly | Not mentioned directly | Not mentioned | Not mentioned | Not mentioned | Not mentioned | Not mentioned | Not mentioned | Not mentioned |
| Sauder et al., 2022 | Surgeons | Surgeon burnout rates were found to be highest among general surgery trainees (20%-95%). Burnout among general surgery attendings ranged from 25% to 44%. Those most likely to experience burnout were younger and female. High rates of surgeon burnout were reported among all surgical specialties; however, these rates were lower than those of general surgeons. | Not mentioned directly but the correlation of the variables is discussed | Not mentioned directly but the correlation of the variables is discussed | Not mentioned directly | Not mentioned | Not mentioned directly | Not mentioned directly | Not mentioned directly | Not mentioned directly | Not mentioned | Not mentioned | Not mentioned | Not mentioned | Not mentioned | Not mentioned | Not mentioned |
| Aymerich et al., 2022 | Healthcare workers | The pooled prevalence of burnout symptoms was 0.37 (95% CI 0.31–0.42). Prevalence varied from 0.22 with Mini-Z to 0.53 with CBI. In MBI, emotional exhaustion was the most deteriorated area among the sample. Sensitivity analyses and meta-regressions revealed no statistically significant differences regarding age, gender. | The pooled prevalence of anxiety was 0.42 (95% CI 0.35–0.48). Again, prevalence varied substantially depending on the scale used, from 0.34 with BAI to 0.68 with STAI-S. Sensitivity analyses and meta-regressions did not show statistically significant differences regarding age, gender. | The pooled prevalence of depression was 0.33 (95% confidence intervals [CI] 0.28–0.38). Prevalence varied widely depending on the scale used, from 0.53 with PHQ-2/4 to 0.26 with CES-D. | The pooled prevalence of post-traumatic symptoms was 0.32 (95% CI 0.26–0.37). Prevalence varied from 0.20 with PCL-C to 0.38 with IES-R. No statistical statistically significant differences regarding age, gender, or NOS score were found in meta-regressions. Sensitivity analyses found a statistically significant lower prevalence of post traumatic symptoms in Asia (0.29; 95% CI 0.18–0.34) compared to North America (0.41; 95% CI 0.34–0.48). | The pooled prevalence of acute stress was 0.40 (95% CI 0.32–0.47). Prevalence varied from 0.26 as measured with DASS-21 to 0.62 with PSS. Again, sensitivity analyses and meta-regressions revealed no statistically significant differences regarding age, gender, | Not mentioned directly | Not mentioned directly | The pooled prevalence of insomnia was 0.42 (95% CI 0.36–0.48). Sensitivity analyses and meta-regressions revealed no statistically significant differences regarding age, gender. | Not mentioned directly | Not mentioned directly | Not mentioned directly | Not mentioned directly | Not mentioned directly | Not mentioned | Not mentioned | Not mentioned |
| Saade et al., 2022 | doctors, nurses, social workers, psychologists, psychiatrists, midwives, occupational therapists, speech pathologists, laboratory and X-ray technicians, community health workers, physical therapist, and eldercare workers | Not mentioned directly but the correlation of the variables is discussed | Not mentioned directly but the correlation of the variables is discussed | Depression prevalence rate varied between 2.5 and 91.30% (M =27.23%, Mdn=23.28%). Those depression prevalence rates need to be interpreted with caution due to the large heterogeneity in the defnition of depression, measurement tool, whether the researchers evaluated depression symptoms, antidepressant use, or a clinical depression diagnosis.The two most frequently reported professions were nurses and doctors with 73.83% and 30.84% of studies including nurses and doctors in their sample. | Not mentioned directly but the correlation of the variables is discussed | Not mentioned | Not mentioned | Not mentioned directly but the correlation of the variables is discussed | Not mentioned directly but the correlation of the variables is discussed | Not mentioned | Not mentioned | Not mentioned | Not mentioned | Not mentioned | Not mentioned | Not mentioned | Not mentioned |
| Musker et al., 2024 | Nurses and midwives | The mean difference for the “burnout” subscale was 3.12 [95 % CI = 0.20, 6.03, Z = 2.09, P = 0.04]. After receiving the intervention, the participant’s burnout scores were reduced significantly. The scores were statistically significant after receiving the intervention. | Not mentioned | Not mentioned | The traumatic stress mean difference was 3.41 [95 % CI = 0.25, 6.56, Z = 2.11, P = 0.03] | Not mentioned | Not mentioned directly | Not mentioned | Not mentioned | Not mentioned | Not mentioned | Not mentioned | Not mentioned directly | Not mentioned | Not mentioned | Psychoeducational; Clinical supervision session; Mindfulness based stress reduction; Communication skills,  Approaches to conflict; Education programme and Peer support; Group cognitive behavioral therapy | Not mentioned |
| Munhoz et al., 2022 | Healthcare workers | There was also a significant improvement for burnout in each participant (p<0.05). | There were significant differences for the reduction of anxiety after 10 sessions. The group with semi-permanent needles reached a large effect and 17% reduction. | Not mentioned | Not mentioned | Not mentioned | Not mentioned directly but the correlation of the variables is discussed | Stress was reduced (p<0.05). There were significant differences between the stress mean values in the second evaluation after 12 appointments (LSS*2) (F=21.92/p=0.000) an in the 30-day follow-up (p.e). | Not mentioned | Not mentioned directly but the correlation of the variables is discussed | Not mentioned | Not mentioned | Not mentioned directly | Not mentioned directly | Not mentioned | Auriculotherapy | Not mentioned |
| Ottisova et al., 2022 | Healthcare workers | No significant changes with 1 single session in burnout symptoms | Authors found that single session interventions significantly reduce anxiety and depression symptoms 1 week after the session. But there were not control group | There were no statistically significant or reliable changes in depressive symptoms with multisession interventions | The authors reported statistically significant reductions in PTSD symptoms at 90-day FUP with 1 single sessions and also in multisession interventions | There were no statistically significant or reliable changes in alcohol consumption with multisessions. | Not mentioned directly | Positive changes with 1 single session in acute stress symptoms | In comprehensive intervention (CBT + trainning) statistically significant improvements in sleep quality, with a large effect size, but, this finding did not constitute reliable change, and staff remained symptomatic above the clinical cutoff. | Not mentioned | Not mentioned | Not mentioned | Not mentioned | Not mentioned | Not mentioned | Psychosocial single and multiple session based on CBT | Not mentioned |
| Wang et al., 2023 | Nurses | There was a improvement en IG compared with CG but, no significant effect of mindfulness-based interventions (MBIs) on any dimension of burnout was found at long-term follow-up. | At post-intervention, the results did not indicate any significant effect of MBIs on anxiety | At post-intervention, the results did not indicate any significant effect of MBIs on depressive symptoms (SMD = −0.24, P = 0.13, 95% CI: −0.55, −0.07). | Not mentioned directly but the correlation of the variables is discussed | Not mentioned | Not mentioned directly | In terms of stress reduction, large effect sizes were observed both immediately post-intervention (SMD = −0.81, P < 0.01, 95% CI: −1.11, −0.52) and at the 3-month follow-up (SMD = −0.69, P < 0.01, 95% CI: −1.08, −0.31), with the latter effect size being moderate in size. | Not mentioned directly | Not mentioned directly | Not mentioned directly | Not mentioned directly | Not mentioned | Not mentioned | Not mentioned | Mindfullnes intervention | Not mentioned |
| Zhou et al., 2024 | Healthcare workers | The meta-analysis results showed that the pooled MD was -6.13 (95% CI: -16.68, 4.43) for MBI Emotional, 5.04 (95% CI: -3.25, 13.33) for the MBI Personal Accomplishment, and -1.68 (95% CI: -6.50, 3.13) for MBI Depersonalization. | The heterogeneity analysis showed significant heterogeneity among the studies (I2=66%, p=0.03), so a random-effects model was employed for the analysis. The meta-analysis results indicated a pooled MD of -0.53 (95% CI:-1.42, 0.37) for GAD-7. | Not mentioned | Not mentioned | Not mentioned | Not mentioned | The meta-analysis results indicated a pooled MD of 0.13 (95% CI: -0.39, 0.65) for post-intervention PSS-4. For the difference in PSS before and after the intervention, the heterogeneity analysis showed significant heterogeneity among the studies (I2=85%, p<0.01), so a random-effects model was employed for the analysis. The meta-analysis results indicated a pooled MD of -0.44 (95% CI: -2.65, 1.76) for the difference in PSS before and after intervention. | Not mentioned | Not mentioned | Not mentioned | Not mentioned | Not mentioned | Not mentioned | Not mentioned | Mindfullnes intervention | Not mentioned |
| Kang et al., 2022 | Nurses | MBIs significantly decreased psychological distress such as burnout, compared with CG | Compared with controls (wait-list, usual care, and active control conditions) A random-effects meta-analysis showed that the MBIs significantly decreased psychological distress such as anxiety. Specifically, MBIs showed a beneficial effect on anxiety (SMD = −0.37; 95% CI, −0.05 to −0.16, I2 = 15.2%. n = 7),. | Results on depression (SMD =−0.46; 95% CI, −0.85 to −0.07; I2 = 47.6%; n=3), showed beneficial efects also | Not mentioned | Not mentioned | MBIs did not show a significant effect on Job related outcomes such as job satisfaction | Results showed an improvement on stress (SMD = −0.61; 95% CI, −1.03 to −0.19; I2= 70.0%; n = 4). | Not mentioned directly | MBIs significantly showed a small beneficial effect on QoL (SMD = 0.28; 95% CI, 0.11 to 0.46; I2= 0.00%; n=8) | Not mentioned directly | Not mentioned | Not mentioned | MBIs did not show a significant effect on Job related outcomes such as job control, workability, and caring efficacy | Not mentioned | Mindfullnes intervention | Not mentioned |
| Karo et al., 2023 | Nurses | Not mentioned directly | The effect of mindfulness-based interventions on reducing anxiety among nurses. The pooled SMD was 0.06 (95% CI: −0.14 to 0.25; p= 0.001, I 2= 9.33%), showing that there was no significant difference in anxiety level between the participants assigned to the intervention and control groups. | The pooled SMD was −0.42 (95% CI: −0.78 to −0.06; p= 0.02, I2= 64.57%), indicating that that mindfulness-based interventions significantly reduced depression symptoms among nurses. | Not mentioned | Not mentioned | Not mentioned directly | The pooled SMD was −0.50 (95% CI: −0.82 to −0.18; p< 0.001, I2= 54.08%) indicating that the mindfulness-based interventions significantly reduced stress among nurses. | Not mentioned | Not mentioned | Not mentioned | Not mentioned | Not mentioned | Not mentioned | Not mentioned | Mindfullnes intervention | Not mentioned |
| Yang et al., 2023 | Healthcare workers | Not mentioned | The results of forest plots showed that the web-based mindfulness-based interventions significantly reduced anxiety (SMD=–0.63, 95% CI –0.96 to –0.31, P<.001, I2=87%) | The results of forest plots showed that the web-based mindfulness-based interventions significantly reduced depression (SMD=–0.52, 95% CI –0.77 to –0.26, P<.001, I2=75%) | Not mentioned | Not mentioned | Not mentioned directly | The results of forest plots showed that the web-based mindfulness-based interventions significantly reduced stress (SMD=–0.20, 95% CI –0.35 to 0.05, P=.01, I2=58%) | Not mentioned | Not mentioned | Not mentioned | Not mentioned | Not mentioned | Not mentioned | Not mentioned | Mindfullnes intervention | Not mentioned |
| Zhang et al., 2024 | Nurses | Not mentioned directly | The results showed a decrease in the SAS scale scores among the art therapy intervention group in comparison with the control group [MD = −1.05, 95% CI (−1.83, −0.27), p< .001] | The analysis revealed a moderate level of heterogeneity among the studies (p< .001, I2= 96%), thus necessitating the utilization of a random effects model. Significant reductions in depression scores were observed in the art therapy groups in comparison with the control groups [MD = −8.01, 95% CI = (−10.18, −5.85), p< .001. | Not mentioned | Not mentioned | Not mentioned directly | The findings suggested that art therapy was associated with significantly lower pressure levels in comparison with the CG, indicating a  reduction in nurses' stress levels. However, the final results were not statistically significant [MD = −5.15, 95% CI (−10.71, −0.42), p= 0.07 | Not mentioned | Not mentioned | Not mentioned | Not mentioned | Not mentioned | Not mentioned | Not mentioned | Art therapy intervention | Not mentioned |
| Lim et al., 2022 | Nurses | Positive correlations were found among variables sucha as burnout(r=0.437, Z=9.44, p< 0.001) | Not mentioned | Not mentioned | Not mentioned | Not mentioned | Negative correlations were found among job satisfaction (r=-0.311, Z=-7.01 , p= 0.07) | The results showed a positive correlations among insufficient job control (r = 0.483, Z = 8.37, p < 0.001) with job stress in nurses. | Not mentioned | Not mentioned | Not mentioned | Not mentioned | Not mentioned | Not mentioned | Not mentioned | Not mentioned | Not mentioned |
| Cheng et al., 2022 | Healthcare workers | Not mentioned | Not mentioned | Not mentioned | Not mentioned | Not mentioned | Not mentioned directly | Not mentioned directly but the correlation of the variables is discussed | Not mentioned | Not mentioned | Not mentioned | Not mentioned | Not mentioned | Not mentioned directly but the correlation of the variables is discussed | Not mentioned | Not mentioned | Not mentioned |
| Papazian et al., 2023 | Physicians and nurses | The prevalence of high-level burn out ranged from 0.15 to 0.71 across ICU physicians with a high level of burnout (random efects model, proportion (prevalence 0.41, range 0.15–0.71, 95% CI [0.33; 0.5], I2 97.6%, 95% CI [96.9%; 98.1%]). The prevalence of high-level burnout ranged from 0.14 to 0.74 across ICU nurses were presenting with burnout (random efects model, proportion (prevalence 0.44, range 0.14–0.74, [95% CI 0.34; 0.55], I2 98.6% 95% CI [98.4%; 98.9%]). The analysis of the 20,723 included ICU professionals revealed that the prevalence of a high level of burnout was not diferent (p=0.63) between ICU physicians (0.41 [95% CI, 0.33; 0.5] and ICU nurses 0.44 [95% CI, 0.34; 0.55]. However, the proportion of ICU professionals with a high level of emotional exhaustion was higher in ICU nurses than in ICU physicians (0.42 [95% CI, 0.37; 0.48] and 0.28 [0.2; 0.39], respectively, p=0.022) | Not mentioned | Not mentioned | Not mentioned | Not mentioned | Not mentioned | Not mentioned | Not mentioned | Not mentioned | Not mentioned | Not mentioned | Not mentioned | Not mentioned | Not mentioned | Not mentioned | Not mentioned |
| Athe et al., 2023 | Healthcare workers | Not mentioned | The results indicate that the prevalence rate of anxiety among HCWs in India during the COVID-19 pandemic ranges from 9.8% to 73.4%, with a pooled estimate of 42.87% (95%CI: 30.26-55.49). | The results indicate that the prevalence rate of depression among HCWs in India during the COVID-19 pandemic ranges from 7.3% to 72%, with a pooled estimate of 35.4% (95%CI: 24.46-46.33). | Not mentioned | Not mentioned | Not mentioned | The meta-analysis of these studies showed that the overall prevalence rate of stress among HCWs in India is 50.38% (95%CI: 22.57-78.19). | Not mentioned directly but the correlation of the variables is discussed | Not mentioned | Not mentioned | Not mentioned | Not mentioned | Not mentioned | Not mentioned | Not mentioned | Not mentioned |
| Jiaru et al., 2023 | Nurses | Not mentioned directly | Not mentioned directly | Not mentioned | Not mentioned | Not mentioned | Not mentioned | The results of meta-analysis showed that the incidence of low-level stress was 0.21% (95% CI [0.18–0.24]), the incidence of medium-level stress was 0.46% (95% CI [0.33– 0.58]), and the incidence of high-level stress was 0.32% (95% CI [0.22–0.41]). | Not mentioned | Not mentioned | Not mentioned | Not mentioned | Not mentioned directly | Not mentioned | Not mentioned | Not mentioned | Not mentioned |
| Lee et al., 2023 | Nurses | The pooled analysis showed that intervention could statistically alleviate burnout (SMD=− 0.654, CI=− 1.584, 0.277, p<0.01, I2=94.8%). For the articles that used the MBI, the pooled analysis showed that intervention could statistically alleviate burnout (SMD=− 0.707, CI=− 1.829, 0.414, p<0.01, I2=87.5%). | Not mentioned directly | Not mentioned | Not mentioned | Not mentioned | Not mentioned | Not mentioned | Not mentioned | Not mentioned | Not mentioned | Not mentioned | Not mentioned directly | Not mentioned | Not mentioned | Mindfulness, yoga, CBT, workplace mutual program, art therapy, violence coping program, empowerment programe, psychodrama based psychological program, accelerated recovery programe, active intervention and regular management, | Not mentioned |
| Teng et al., 2023 | Nurses | The pooled mean score of mental workload was 68.07 (95%CI:64.39–71.75) | Not mentioned | Not mentioned | Not mentioned | Not mentioned | Not mentioned | Not mentioned | Not mentioned | Not mentioned | Not mentioned | Not mentioned | Not mentioned directly | Not mentioned | Not mentioned | Not mentioned | Not mentioned |
| Huang et al., 2024 | Healthcare workers | The prevalence of burnout, with the combined prevalence rate in health workers being 47% (95% CI, 38%-55%, Fig 2). Individual study estimates ranged from 12% to 73% and there was evidence of high between-study heterogeneity (I2 = 99.81%, p<0.001). | The prevalence of anxiety disorders, with the combined prevalence rate of anxiety among health workers being 38% (95% CI, 35–41%). Individual study estimates ranged from 6% to 90% and there was evidence of high heterogeneity between studies (I2 = 99.67%, p<0.001). | The prevalence of depression, with the combined prevalence rate of depression among healthcare workers being 34% (95% CI, 30–38%, Fig 4). Estimates for a single study ranged from 4% to 91%, and there was evidence of high heterogeneity between studies (I2 = 99.81%, p<0.001). | The prevalence of post-traumatic stress disorder (PTSD), with the combined prevalence rate of PTSD among healthcare workers being 26% (95% CI, 22–29%). Individual study estimates ranged from 2% to 67% and there was evidence of high between-study heterogeneity (I2 = 99.64%, p<0.001). | Not mentioned | Not mentioned | The prevalence of acute stress disorder, with the combined prevalence rate of acute stress disorder among healthcare workers being 30% (95% CI, 29–31%) | Not mentioned | Not mentioned | Not mentioned | Not mentioned | Not mentioned | Not mentioned | Not mentioned | Not mentioned | Not mentioned |
| Le Huu et al., 2022 | Physicians | Not mentioned | Not mentioned | Not mentioned | Not mentioned | The overall standardized pooled mean score was 62.1 [56.2–67.9] (Q=31.0) versus 50.5 [44.8–56.1] (Q =18.3) for studies using six items. For more details, the pooled effort score was 63.8 [53.2–74.4] For the group using the three items version, 61.7 [51.7–71.7] for the four items version, and 62.4 [49.4–75.5] for the five items version. | Not mentioned | Not mentioned | Not mentioned | Not mentioned | Not mentioned | Not mentioned | Not mentioned | Not mentioned | Not mentioned | Not mentioned | Not mentioned |
| Chen et al., 2022 | Healthcare workers in general | Not mentioned | The results showed that the overall prevalence of anxiety was 0.43, with a 95% CI (0.36, 0.50), P < 0.001. | The results showed that the overall prevalence of depression was 0.45, with a 95% CI (0.37, 0.52), P < 0.001. | Not mentioned | Not mentioned | Not mentioned | Not mentioned | Not mentioned | Not mentioned | Not mentioned | Not mentioned | Not mentioned | Not mentioned | Not mentioned | Not mentioned | Not mentioned |
| Colin et al., 2023 | Health care professionals in general | For mental workload in the operating room, the best results were also significant with Mozart’s music, however, only for nurse anesthetists, and at a low level of 55–60 dB compared to 75–80 dB (P < 0.01).27 For the MM study considering the risk of burnout (Maslash Burnout Inventory (MBI) questionnaire), the impact was significant for emotional exhaustion (P = 0.004), but not for depersonalization or and impairment of personal achievement. | One study involved the sound level with the best State–Trait Anxiety Inventory (STAI-S) score for 55–60 dB compared to 75–80 dB (P < 0.001).27 When biological parameters were assessed to evaluate active MM with Mahamantra songs, significant results were reported for serum cortisol and salivary α-amylase. Two studies investigated the anxiety outcome.26,27 Both receptive passive MM (STAI-S questionnaire, P < 0.01) and active MM with instruments (questionnaire HADS) showed significant decreased anxiety compared to respective control groups (effect size Cohen’s d = 0.83). Among the three types of recorded music, the best mean rating score was for Mozart’s music (P < 0.01). | Not mentioned | Not mentioned | Not mentioned | Not mentioned | Job Stress Questionnaire (JSS) or specific-study questionnaires yielding significant results for one MT study and two MM studies. Only statistically trend for change over time between groups (P = 0.07) was reported in a MT study, with the best score for customized PLs compared to algorithmic music | Not mentioned | Not mentioned | Not mentioned | Not mentioned | Not mentioned | Not mentioned | Not mentioned | Music based interventions | Not mentioned |
| Khatatbeh et al., 2022 | Nurses | The results found a negative correlation between nurses’ burnout and their QOL or professional QOL | Not mentioned | Not mentioned | Not mentioned | Not mentioned | Not mentioned | Not mentioned | Not mentioned | The results found that nurses’ QOL was negatively correlated with emotional exhaustion and depersonalization, and positively with personal accomplishment | Not mentioned | Not mentioned | Not mentioned | Not mentioned | Not mentioned | Not mentioned | Not mentioned |
| Xia et al., 2023 | Nurses | The results found that gender was not a significant predictor of job burnout. Education level was significantly associated with job burnout. Relevant studies have shown that nurses with bachelor’s degrees have the highest level of job burnout among all academic groups. | Not mentioned | Not mentioned | Not mentioned | Not mentioned | Not mentioned | Not mentioned | Not mentioned | Not mentioned | Not mentioned | Not mentioned | Not mentioned | Not mentioned | Not mentioned | Not mentioned | Not mentioned |
| Zhang et al., 2024 | Healthcare workers in general | Not mentioned | Not mentioned | Not mentioned | The overall prevalence of Post-traumatic stress disorder among Chinese healthcare workers during the COVID-19 epidemic was 29.2% (95% CI: 20.7% to 33.7%). | Not mentioned | Not mentioned | Not mentioned | Not mentioned | Not mentioned | Not mentioned | Not mentioned | Not mentioned | Not mentioned | Not mentioned | Not mentioned | Not mentioned |
| Harris et al., 2023 | Healthcare workers in general | Not mentioned | The results showed that 46.4% participants were significantly more anxious, 35.7% reporting increased anger, and 28.6% reported feeling significantly less outgoing. | Not mentioned | Not mentioned | Not mentioned | Not mentioned | Not mentioned | Not mentioned | Not mentioned | Not mentioned | Not mentioned | Not mentioned | Not mentioned | Not mentioned | Not mentioned | Not mentioned |
| Algamdi., 2022 | nurses | For the meta-analysis of the prevalence of low, medium to high BO and STS, six studies were included. The meta-analysis with 95% confidence intervals had the lowest prevalence of CS (22.89%) (10.77–37.7). For medium to high BO and STS, the prevalence rates were 62.76% (47.30–77.5) and 66.84% (47.15–83.98), respectively. | Not mentioned | Not mentioned | Not mentioned | Not mentioned | Not mentioned | Not mentioned | Not mentioned | The results of the Egger linear regression test were statistically significant (p > .05). This shows there was no publication bias or small study effects in the meta-analysis. The I2 heterogeneity analysis showed 99.88% for CS, 99.85% for BO, and 99.9% for STS | Not mentioned | Not mentioned | Not mentioned | Not mentioned | Not mentioned | Resilience programme and mobile application, nurse-led interventions, and mindfulness-based interventions | Not mentioned |
| Alberque et al., 2022 | Healthcare workers in general | Not mentioned | Not mentioned | Not mentioned | The overall prevalence of PTSD was 14% in HCWs (95CI 10 to 17%). More specifically, the prevalence was 16% (8 to 24%) during the epidemic, 19% (16 to 22%) between 1 to 6 months after the end of the epidemic, and 8% (4 to 13%) more than 1 year after the epidemic. | Not mentioned | Not mentioned | Not mentioned | Not mentioned | Not mentioned | Not mentioned | Not mentioned | Not mentioned | Not mentioned | Not mentioned | Not mentioned | Not mentioned |
| de Vargas et al., 2023 | nurses | The results showed that the predominant symptoms were burnout (52.1%, 95% CI: 37.1%, 88.8%; I2=98.5%, p<0.001) | The highest prevalence of anxiety symptoms was 55.2% (IC 95%: 47.2%, 62.8%; I2=88.5%, p<0.001), 53.2%; I2=98.6%, p<0.001). | The highest prevalence of depression 58.5% (IC 95%: 51.4%, 65.3%; I2=97.1%, p<0.001) | The prevalence of PTSD was 65.9% (IC 95%: 62.6%, 69%; I2=0%, p<0.001), | The results found a prevalence rates of fear (52.1%, 95% CI: 30.1%, 73.3%; I2=98.1%, p<0.001), | Not mentioned | Not mentioned | The results showed a prevalence of insomnia (46.9%, 95% CI: 31.8%, 62.5%; I2=97.7%, p<0.001) | Not mentioned | Not mentioned | Not mentioned | Not mentioned | Not mentioned | Not mentioned | Not mentioned | Not mentioned |
| You et al., 2022 | nurses | Not mentioned | Not mentioned | Not mentioned | Not mentioned | Not mentioned | Not mentioned | Not mentioned | Not mentioned | Not mentioned | Not mentioned | Not mentioned | Not mentioned | The total score of nurses’ work alienation was 35.43 [95%CI (31.827, 39.040)], which was in the middle level | Not mentioned | Not mentioned | Not mentioned |
| Bekelepi et al., 2022 | nurses | Regarding to burnout resouls found in some studies that prior intervention- total burnout score IG 71.13↑ CG 66.28 1-month post intervention IG 63.15 CG 67.93↑ 3-month post intervention IG 64.88 CG 68.74↑ 6-month post intervention IG 66.15 CG 69.99↑ | Following the implementation of the intervention, the psychotherapy group had an effect on participants’ anxiety about possible violent incidents | After participation on the interventions the depression that results from such anxiety seemed to have been alleviated. | Not mentioned | Not mentioned | Job satisfaction: IG 3.33↑ versus CG3.16 Potentiality: IG3.22↔CG3.15 | Results showed a significant impact on stress reduction and how nurses cope with challenges using positive coping mechanisms after participating in the intervention | Not mentioned | Not mentioned | Not mentioned | Not mentioned | Not mentioned | Not mentioned | Not mentioned | Educational support, Psychological support, Adaptive coping, and Interpersonal skills | Not mentioned |
| Claponea et al., 2022 | Physicians | The overall burnout ranged from 14.7% to 90.4%. | Anxiety and COVID-19-related burnout were significantly associated with the physician’s age, gender, and years in practice. | Burnout was positively associated with a history of depression | Not mentioned | Not mentioned | Not mentioned | Not mentioned | Some studies identified an increase rate of consumption of pills (for stress and sleep related problems) | Symptoms of moderate-to-severe depression and anxiety have a negative impact on doctors’ quality of life, so anxious physicians have had a lower quality of life compared to the non-anxiety group | Not mentioned | Not mentioned | Not mentioned | Not mentioned | Not mentioned | Not mentioned | Not mentioned |
| Chen et al., 2022 | Doctors | Not mentioned | Not mentioned | Not mentioned | Not mentioned | Not mentioned | The meta-analysis showed that the overall job satisfaction score of village doctors was 3.1858 (total score: 5.00), 95% CI: 2.9675–3.404, which represented the level of “neither satisfied nor dissatisfied.” | Not mentioned | Not mentioned | Not mentioned | Not mentioned | Not mentioned | Not mentioned | Not mentioned | Not mentioned | Not mentioned | Not mentioned |
| Sulosaari et al., 2022 | nurses | Five studies found that mindfulness-based programmes reduce nurses' burnout | Not mentioned | Not mentioned | Not mentioned | Not mentioned | Not mentioned | The results determined that mindfulness-based programmes decrease the stress level of nurses | Not mentioned | Not mentioned | Not mentioned | Four studies found mindfulness-based trainings to be efficient in improving the level of self compassion among nurses | Not mentioned | Not mentioned | Not mentioned | Mindfulness intervention | Not mentioned |
| Yoo et al., 2022 | nurses | Not mentioned | Not mentioned | Not mentioned | Not mentioned | Not mentioned | Not mentioned | Six studies reported significant positive effects of CBT on stress among healthcare workers. | Two studies reported significant positive effects of CBT on insomnia among healthcare workers. | Not mentioned | Not mentioned | Not mentioned | Not mentioned | Three studies reported significant positive effects of CBT on work related outcomes among healthcare workers | Not mentioned | CBT Interventions | Not mentioned |
| Sheng et al., 2022 | Doctors | The pooled burnout rate showed 37%, 28%, and 26% of general GPs suffer from high emotional exhaustion (EE), high depersonalization (DP), and low personal exhaustion (PA), respectively. | Not mentioned | Not mentioned | Not mentioned | Not mentioned | Not mentioned | Not mentioned | Not mentioned | Not mentioned | Not mentioned | Not mentioned | Not mentioned | Not mentioned | Not mentioned | Not mentioned | Not mentioned |
| Johns et al., 2022 | Doctors | Not mentioned | The pooled prevalence of anxiety for the 30 included studies was 25.8% (95% CI 20.4-31.5%), with a similarly high degree of heterogeneity (I2= 99.190%), | The pooled prevalence of depression for the 26 included studies was 20.5% (95% CI 16.0-25.3%), with a high degree of heterogeneity (I2= 98.931%), | Not mentioned | Not mentioned | Not mentioned | Not mentioned | Not mentioned | Not mentioned | Not mentioned | Not mentioned | Not mentioned | Not mentioned | Not mentioned | Not mentioned | Not mentioned |
| Wang et al., 2024 | Nurses | Occupational burnout was found to have a negative impact on nurses' PTG in two studies | Not mentioned | Not mentioned | Not mentioned | Not mentioned | The results found that job satisfaction were positively associated with PTG among nurses in different studies | Not mentioned | Not mentioned | Not mentioned | Not mentioned | Not mentioned | Not mentioned | Social and organizational-related factors, such as social support and receiving systematic training were found to have a positive relationship with PTG among nurses in multiple studies. | Not mentioned | Not mentioned | Not mentioned |
| Bai et al., 2023 | Doctors | The pooled prevalence of burnout was 61.7% (95% confidence interval (CI), 48.6–73.2%; I2= 96.3%). | Not mentioned | Not mentioned | Not mentioned | Not mentioned | Not mentioned | Not mentioned | Not mentioned | Not mentioned | Not mentioned | Not mentioned | Not mentioned | Not mentioned | Not mentioned | Not mentioned | Not mentioned |
| Long et al., 2023 | Dentist | The overall prevalence of burnout among dentists was 13% (95%CI: 6–23) | Not mentioned | Not mentioned | Not mentioned | Not mentioned | Not mentioned | Not mentioned | Not mentioned | Not mentioned | Not mentioned | Not mentioned | Not mentioned | Not mentioned | Not mentioned | Not mentioned | Not mentioned |
| Ma et al., 2023 | Nurses | According to the most common criteria used to define burnout (the score ≥ 27 for EE, ≥10 for DP and ≤ 33 for PA), the pooled mean scores indicate that the average oncology nurse was at a moderate level of EE and DP and a low level of PA. This study found that 36.4% of oncology nurses were reporting high levels of EE, 28.26% experienced high levels of DP and 28.69% perceived low levels of PA. | Not mentioned | Not mentioned | Not mentioned | Not mentioned | Not mentioned | Not mentioned | Not mentioned | Not mentioned | Not mentioned | Not mentioned | Not mentioned | Not mentioned | Not mentioned | Not mentioned | Not mentioned |
| Xiong et al., 2022 | Healthcare workers in general | Not mentioned | Resulting from 18 studies with 34,793 participants, the overall prevalence of moderate to severe anxiety was estimated as 17% (95% CI 13–21%) | The prevalence of moderate to severe level of depression was estimated as 15% (95% CI 13–16%) (k=13n = 48,621) | The overall prevalence was 27% (95% CI 16–38%) with substantial heterogeneity (I2 = 99.8%, p = 0.02). | Not mentioned | Not mentioned | Not mentioned | The overall prevalence of moderate to severe sleep disturbance was estimated as 15% (95% CI 7 23%, k = 5, n = 5,711) with substantial heterogeneity (I2 = 99.1%, p = 0.01) | Not mentioned | Not mentioned | Not mentioned | Not mentioned | Not mentioned | Not mentioned | Not mentioned | Not mentioned |
| PTSD: Posttraumatic stress disorder; PSQI: Pittsburgh Sleep Quality Index; STAI: State-trait anxiety inventory; BDI:Beck depression inventory; PTGI: Posttraumatic Growth Inventory; IES-R: Impact of Events Scale-Revised; R-Cope: R-COPE Inventory; NEO-FFI: revised Costa and McCrae; BCI: Basic Character Inventory; GHQ: General Health Questionnaire; IES/IES-R/IES-15: The Impact of Event Scale/-Revised; MBI/MBI-HSS: Maslach Burnout Inventory/MBI-Human Services Survey; HSE: Health and Safety Executive Job Stress Questionnaire; AWSQ: Ambulance Work Stressors Questionnaire; HADS: Hospital anxiety and depression scale; PCL-C: Posttraumatic stress disorder checklist-civilian version; SF-36: short form Health survey-36; SOP-2: Optimism-Pessimism-2 Scale; OSSS: Oslo Social Support Scale; PHQ-4: Patient Health Questionnaire 4; CAR: Cortisol awakening response; PSS: Perceived stress scale; CBI: Copenhagen Burnout Inventory; CSD: Consensus Sleep diary; DTS: Davidson Trauma scale for PTSD; AUDIT: Alcohol Use Disorders Identification Text; QEAW: Questionnaire on the Experience and Assessment of Work; CIS: Checklist of Individual Strength.; GAD-7: Generalised Anxiety Disorder Assessment-7; DASS-21: Depression, Anxiety and Stress Scale-21; NQoL-SAT-P: Nurses Quality of Life Scale−Satisfaction Profile; SAS: Zung Self-Rating Anxiety Scale; BAI: Beck Anxiety Inventory; CAS: Coronavirus Anxiety Scale; SARSQ: Stanford Acute Stress Reaction Questionnaire; ISI: Insomnia Severity Index; SQS: Sleep Quality Scale; Mini-Z: Mini-Z Burnout Survey; PANAS: Positive and Negative Affect Schedule; CD-RISC: Connor–Davidson Resilience Scale; MMSS: The McCloskey/Mueller Satisfaction Scale; MMSS: The McCloskey/Mueller Satisfaction Scale; SAS: The self -rating anxiety scale; WHOQOL-BREF: World Health Organization Quality of Life-BREF; WAI: Workability Index Scale; ENSS: Expanded nursing work stress scale; HSS-35: The hospital stress scale; OLBI: Oldenburg Burnout Inventory; PROMIS: National Institution of Mental Health; HAM-D: Hamilton Depression Scale; ISR: The self-report questionnaire; ASDS: Acute Stress Disorder Scale; DTS-8: The Davidson Trauma Scale; IES-R: The 22-item Impact of Event Scale-Revised; MASI-R: Maugeri Stress Index-Revised; IWS: Lee’s Index of Work Satisfaction; POMS: Profile Mood State;BMS: The 10-item Burnout Measure Short Version; CFDT: The Compassion Fatigue and Satisfaction Self-Test; FCV-19S: The Fear of COVID-19 scale; UWES: Utrecht Work Engagement Scale; PANAS: Positive and Negative Affect Schedule; GSES: The General Self-Efficacy Scale | | | | | | | | | | | | | | | | | |
