# Supplementary material for: Strategies to support the mental health and well-being of health and care workforce: a rapid review of reviews
Source: Front Med (Lausanne). 2025 Mar 19;12:1530287. doi: 10.3389/fmed.2025.1530287 (PMC11961965; doi:10.3389/fmed.2025.1530287)
Supplement: Supplementary file 3 [file Table_3.docx]

| **Reference** | **Sample** | **Sample Size** | **Country** | **Mental Health Burden** | | | | | |
| --- | --- | --- | --- | --- | --- | --- | --- | --- | --- |
|  |  |  |  | **Burnout** | **Anxiety** | **Depression** | **PTSD** | **Others** | **Aims and key themes** |
| **Thielmann et al., 2022. Germany** | Paramedics   Emergency medical services professionals  Emergency medical technicians  Emergency medical drivers  Emergency physicians | 31.668 | Germany | Prevalence = 8% | Anxiety symptoms < general population | Depression symptoms < general population (for men) | Prevalence = 5.6% | 18.8% (men) and 10.7% (women) used alcohol to cope.   22.8% reported a lifetime suicidal ideation (10.4% of those reporting serious suicidal ideation and 3.1% reporting having previously attempted suicide)  There was a higher prevalence of women who ‘wished they were dead’. | The aim of this review was to analyze the literature on the associations between psychosocial or physical work factors on one hand and the well-being outcomes and job satisfaction on the other hand. The review found a high prevalence of psychological and physical stress among EMS personnel, with burnout and PTSD being the most studied outcomes. Higher qualifications were linked to better health, but age was not always a protective factor. |
| **Egbe et al., 2024** | Surgeons | 11.399 | UK | Substantial incidence of burnout associated with other mental health conditions. | Median percentage = 20% | Median percentage = 24% | Not specified (n/s) | One study found the rate of suicidal thoughts to be 1.5 to 3 times > than the national average, but only 26% of those with suicidal thoughts had sought help, and 60% of those with suicidal ideation were hesitant to seek help due to concerns about their career.   One study found a high level of mental health disorders including drugs abuse. | To review the prevalence of anxiety and depression among surgeons over the past 10 years, to identify factors associated with anxiety and depression in surgeons and discuss potential methods to address their impact. It was found a high prevalence of anxiety (median 20%) and depression (median 24%). Risk factors included female gender, younger age, burnout, and COVID-19-related concerns, while institutional support and social belonging were protective. The study also highlights the need for strategies to reduce anxiety and depression in surgeons. |
| **Nicolakakis et al., 2022** | Nurses   Doctors | n/s | Canada | n/s | Recognition measures of prevention pandemic programmes are associated with 24% ↓ likelihood of anxiety (GAD-7 ≥ 8) compared to not having received recognition measures: AOR (95% CI): 0.76 (0.60–0.97); p = 0.03 | Recognition measures of prevention pandemic programmes are associated with 31% ↓ likelihood of depression (PHQ-9 ≥ 10) compared to not having received recognition measures: AOR (95% CI): 0.69 (0.52–0.90); p = 0.007 | n/s | n/s | To evaluate the effectiveness of organizational and psychosocial work environment interventions to protect healthcare workers’ mental health in an epidemic/pandemic context. Multicomponent programs, including staffing adjustments, shift changes, infection control, and psychological support, showed promise. However, evidence quality of the intervestions found was low due to methodological limitations. |
| **Galanis et al, 2024** | Nurses | 3.730 | Greece | Stronger association between workplace bullying and job burnout in studies with > percentage of females (coefficient beta = 0.01, 95% CI = 0.006 to 0.019, p = 0.001). | n/s | n/s | n/s | n/s | To quantitatively summarize the data on the association between workplace bullying and job stress and the professional quality of life of nurses. It was found workplace bullying moderately correlated with increased job stress (0.34), burnout (0.43), and secondary traumatic stress (0.36), while compassion satisfaction showed a small negative correlation (−0.28). The study also highlights the need for effective interventions to mitigate workplace bullying and improve nurses' professional quality of life. |
| **Zheng et al., 2022** | Doctors | 48.638 | China | Overall prevalence = 75.48%   High burnout = 9.37% | n/s | n/s | n/s | n/s | To analyze surveys measuring the prevalence of burnout among Chinese doctors and reveal the overall prevalence, characteristics, timeline, and factors related to burnout. A meta-analysis of 64 studies (48,638 Chinese doctors) found a high overall burnout prevalence (75.48%), with 9.37% experiencing severe burnout. Emotional exhaustion (48.64%), depersonalization (54.67%), and reduced personal accomplishment (66.53%) were key components. Burnout peaked from 2008 to 2017, then declined from 2018 to 2020 before slightly rising in 2021. Factors like gender, marital status, and professional title influenced burnout levels. |
| **Hiver et al., 2022** | Physicians | 27.903 | France | Rates from 2.5% to 72% | n/s | n/s | n/s | n/s | To assess burnout prevalence rates among physicians practicing in Europe (regardless of their specialty) taking into account the main approaches used to define burnout with the Maslach Burnout Inventory (MBI) tool. Burnout prevalence varied widely, ranging from 2.5% to 72.0%, depending on the definition used. Pooled prevalence was 7.7% (tridimensional), 19.7% (bidimensional), and 43.2% (unidimensional). |
| **Moro et al., 2022** | Dentists | 8.823 | Brazil | Overall prevalence = 13% | n/s | n/s | n/s | n/s | To assess the prevalence of Burnout syndrome in dentists, which was found to be of 13% of prevalence. Emotional exhaustion was the most affected subscale (28%), followed by depersonalization (18%) and reduced personal accomplishment (10%). High levels of emotional exhaustion (25%) and depersonalization (18%) were reported. The study also highlights a significant impact of burnout on dentists, particularly in emotional exhaustion. |
| **Sauder et al., 2022** | Surgeons | n/s | USA | 20% to 95% (general surgery trainees)  25% to 44% (general surgery attendings)  Especially in younger people and female. | n/s | n/s | n/s | n/s | To systematically characterize existing definitions of burnout, evaluate tools to measure burnout, and determine risk factors of surgical burnout. High burnout rates among surgeons were found, especially general surgery trainees (20%-95%) and attendings (25%-44%). Younger and female surgeons were at higher risk. Burnout was consistently characterized by emotional exhaustion, depersonalization, and reduced personal accomplishment, with the Maslach Burnout Inventory being the most used assessment tool. The study highlights the need for institutional support and individual strategies to mitigate burnout in surgery. |
| **Aymerich et al., 2022** | Healthcare workers | 271.319 | Spain | Prevalence = 37% (CI: 31-42) | Prevalence = 42%  (CI: 35-48)   No statistically significant differences regarding age or gender. | Prevalence = 33%  (CI: 28-38) | Prevalence = 32%  (CI: 26-37)  No statistically significant differences regarding age or gender.  Statistically significant lower prevalence of post traumatic symptoms in Asia (29%) compared to North America (41%) | Prevalence of acute stress = 40% (CI: 32-47)  No statistically significant differences regarding age or gender. | This systematic review of 239 studies found high prevalence rates of mental health issues due to COVID-19 exposure: anxiety (42%), acute stress (40%), insomnia (42%), depression (33%), post-traumatic symptoms (32%), and burnout (37%). Meta-regressions indicated lower post-traumatic symptoms in Asia. |
| **Saade et al., 2022** | Doctors  Nurses and Midwives  Social workers  Psychologists  Psychiatrists  Occupational therapists  Speech pathologists   Laboratory and X-ray technicians   Community health workers   Physical therapist   Eldercare workers | 17.437 | Lebanon | n/s | n/s | Prevalence range between 2.5% and 91.30% | n/s | n/s | To provide an overview of the prevalence rate of depression in a wide array of helping professions, and identify work organization conditions that seem to be associated with this depression risk. There were found widely varying depression rates (2.5%–91.3%) in helping professions, with nurses and doctors most frequently studied. Work-related factors linked to depression included job demands, long hours, irregular schedules, low decision authority, lack of social support, job insecurity, and workplace bullying. |
| **Musker et al., 2024** | Nurses and midwives | 3.007 | Australia | Mean difference = 3.12  After receiving the intervention, the participant’s burnout scores were reduced significantly. | n/s | n/s | Mean difference = 3.41 | n/s | To examine the effectiveness of interventions to ameliorate burnout, secondary traumatic stress, and emotional exhaustion in nurses and midwives. The interventios of the this systematic review and meta-analysis targeted burnout, secondary traumatic stress, and emotional exhaustion in nurses and midwives. Studies using the ProQOL scale showed a positive improvement (Z = 2.07, P = 0.04), while those using the MBI reported a stronger effect (Z = 3.13, P = 0.002). The most effective interventions included clinical supervision and personal well-being activities, while managerial and purely educational approaches were less effective. |
| **Munhoz et al., 2022** | Healthcare workers | 860 | Brazil | Significant improvement for burnout in each participant (p<0.05) after auriculotherapy intervention | Significant differences for the reduction of anxiety after 10 sessions. The group with semi-permanent needles reached a large effect and 17% reduction. | n/s | n/s | n/s | To analyze the effectiveness of auriculotherapy, when compared to the control group, placebo or usual treatment for anxiety, stress or burnout in health professionals. Most studies involved nursing teams (66.6%) and used semi-permanent needles (53.3%). Anxiety significantly decreased with semi-permanent needles (CI -8.18, -6.10), magnetic palettes (CI -7.76, -5.54), placebo (CI -5.47, -3.36), and seeds (CI -6.35, -4.05). Stress reduction was observed with semi-permanent needles (CI -37.21, -10.88) and seeds, both with (CI -28.14, -11.70) and without a closed protocol (CI -36.42, -10.76). |
| **Ottisova et al., 2022** | Healthcare workers | Not defined | UK | No significant changes with 1 single session in burnout symptoms | Single session interventions significantly reduced anxiety and depression symptoms 1 week after the session. | No statistically significant or reliable changes in depressive symptoms with multisession interventions | Statistically significant reductions in PTSD symptoms at 90-day follow up with 1 single session and multisession interventions | There were no statistically significant or reliable changes in alcohol consumption with multisessions. | This systematic review analyzed psychosocial interventions for healthcare workers before, during, and after disasters, focusing on PTSD, anxiety, depression, and sleep disturbances. Among 12,198 screened papers, 14 were included. Cognitive behavioral therapy (CBT), both individual and group-based, showed the most reliable improvements in PTSD and anxiety symptoms. However, single-session debriefing and psychological first aid workshops demonstrated limited efficacy. |
| **Wang et al., 2023** | Nurses | 1.165 | China | Improvement in IG compared with CG, but no significant effect of mindfulness-based interventions (MBIs) of burnout was found at long-term follow-up. | No significant effects after MBIs on anxiety | No significant effects after MBIs on depression | n/s | n/s | To systematically evaluate the impact of mindfulness training on nurse’s performance and increase the certainty of existing evidence. In 15 randomized controlled trials with 1,165 participants, the study found moderate effectiveness in reducing stress and significant improvements in burnout, particularly in emotional exhaustion, depersonalization, and personal accomplishment. However, no significant effects were observed for anxiety or depression. While stress reduction was immediate, burnout improvements appeared over time, suggesting mindfulness training as a beneficial tool for long-term well-being among nurses. |
| **Zhou et al., 2024** | Healthcare workers | 1.466 | China | Pooled MD = -6.13 (95% CI: -16.68, 4.43) for MBI Emotional  Pooled MD = 5.04 (95% CI: -3.25, 13.33) for the MBI Personal Accomplishment  Pooled MD = -1.68 (95% CI: -6.50, 3.13) for MBI Depersonalization. | Pooled MD = -0.53 (95% CI:-1.42, 0.37) for GAD-7. | n/s | n/s | n/s | To explore the treatment effects of various intervention methods on the stress, anxiety, and fatigue of medical workers during the 2019 Coronavirus Disease (COVID-19) pandemic. Among 1,466 participants, mindfulness-based therapy was the most common intervention. The meta-analysis found minimal improvements in anxiety, stress, and burnout, with mixed results across subscales of the Maslach Burnout Inventory (MBI). While mindfulness therapy was widely used, its effectiveness remains inconclusive, highlighting the need for further research to optimize mental health interventions for frontline healthcare workers. |
| **Kang et al., 2022** | Nurses | 572 | Korea | MBIs significantly decreased psychological distress such as burnout, compared with CG | MBIs significantly decreased psychological distress such as anxiety.   Specifically, MBIs showed a beneficial effect on anxiety (SMD = −0.37; 95% CI, −0.05 to −0.16, I2 = 15.2%. n = 7),. | MBIs significantly decreased psychological distress such as depression (SMD =−0.46; 95% CI, −0.85 to −0.07; I2 = 47.6%; n=3) | n/s | n/s | To evaluate the psychological effects of mindfulness-based interventions (MBIs) on mental health in nurses. It was found that MBIs significantly reduced psychological distress (SMD = −0.47) and slightly improved well-being (SMD = 0.28). However, MBIs had no significant effect on job-related outcomes. |
| **Karo et al., 2023** | Nurses | 1.372 | Indonesia | n/s | Pooled SMD = 0.06 (95% CI: −0.14 to 0.25; p= 0.001, I 2= 9.33%)   (No significant difference in anxiety level between the participants) | Pooled SMD = −0.42 (95% CI: −0.78 to −0.06; p= 0.02, I2= 64.57%)   (MBIs significantly reduced depression symptoms) | n/s | n/s | To examine the effects of mindfulness-based interventions on reducing stress, anxiety, and depression among nurses. The results showed that MBIs significantly reduced stress (SMD: −0.50) and depression (SMD: −0.42). These findings suggest that MBIs can be effective in alleviating psychological distress among nurses. |
| **Yang et al., 2023** | Healthcare workers | 1.311 | China | n/s | MBIs significantly reduced anxiety (SMD=–0.63, 95% CI –0.96 to –0.31, P<.001, I2=87%) | MBIs significantly reduced depression (SMD=–0.52, 95% CI –0.77 to –0.26, P<.001, I2=75%) | n/s | n/s | To explore whether web-based mindfulness-based interventions continue to have a positive impact on anxiety, depression, and stress among health care workers during the COVID-19 pandemic. A total of 10 randomized controlled studies with 1311 participants were included. Results indicated that web-based mindfulness interventions significantly reduced anxiety (SMD = -0.63), depression (SMD = -0.52), and stress (SMD = -0.20), though the effects were mild and accompanied by high heterogeneity. |
| **Zhang et al., 2024** | Nurses | 1.338 | China | n/s | Decrease in the SAS scale scores among the art GI in comparison with the CG [MD = −1.05, 95% CI (−1.83, −0.27), p< .001] | Significant reductions in depression scores were observed in the art therapy groups GI in comparison with the CG [MD = −8.01, 95% CI = (−10.18, −5.85), p< .001. | n/s | n/s | To evaluate the efficacy of art therapy (AT) in improving the mental health of clinical nurses. A systematic review of 19 RCTs involving 1,338 nurses found that AT significantly reduced anxiety, depression, and perceived stress levels. It also improved positive coping styles and reduced negative coping behaviors. |
| **Lim et al., 2022** | Nurses | N/S | Korea | Positive correlations were found between job stress and burnout (r=0.437, Z=9.44, p< 0.001) | n/s | n/s | n/s | n/s | To analyze job stress in hospital nurses from 2008 to 2018. It was found positive correlations between job stress and factors like insufficient control, personal conflict, and burnout. Negative correlations were found with job satisfaction and personal accomplishment. |
| **Cheng et al., 2022** | Healthcare workers | 17.073 | Singapore | n/s | n/s | n/s | n/s | n/s | To examine the prevalence of low resilience among healthcare professionals and the factors influencing it. Among 41 studies (17,073 participants), the prevalence of low resilience was found to be 26%. The review highlighted that different resilience measures and high stress during the COVID-19 pandemic, particularly in the Middle East, impacted these rates. |
| **Papazian et al., 2023** | Physicians   Nurses | 20.723 | France | Prevalence of high-level burn out = 41% (CI: 33-50) in ICU physicians  Prevalence of high-level burn out = 44% (CI: 34-55) in ICU nurses   Higher level of emotional exhaustion in ICU nurses (42%) than in ICU physicians (28%) | n/s | n/s | n/s | n/s | To estimate the prevalence of high-level burnout in physicians and nurses working in adult ICUs using the Maslach Burnout Inventory (MBI). It was found that 41% of ICU physicians and 44% of nurses experienced high-level burnout, with higher rates observed during the COVID-19 pandemic. Nurses had higher emotional exhaustion than physicians. The study highlights significant heterogeneity in burnout levels, suggesting a need for a consistent definition of burnout to compare and assess interventions effectively. |
| **Athe et al., 2023** | Healthcare workers | 3.420 | India | n/s | Prevalence = 35.4%  (CI: 24.46-46.33) | Prevalence = 34.9%  (CI: 27.33-42.47) | n/s | n/s | To explore the gender perspective of mental health conditions among HCWs and job loss during the pandemic in India. The meta-analysis of 11 studies found high prevalence rates for depression (34.9%), anxiety (35.4%), stress (32.9%), and worry (42.9%) among HCWs, with 16.6% of HCWs experiencing job loss. |
| **Jiaru et al., 2023** | Nurses | 4.293 | China | n/s | n/s | n/s | n/s | n/s | To evaluate the stress level among emergency nurses systematically among 21 cross-sectional studies with 4,293 participants. The meta-analysis found that 46% of emergency nurses experienced moderate stress, 32% had high stress, and only 21% had low stress. |
| **Lee et al., 2023** | Nurses | 1.935 | Korea | Pooled prevalence = 11.2% | n/s | n/s | n/s | n/s | The study analyzed 30 articles on burnout interventions for clinical nurses, with mindfulness being the most common approach. Meta-analysis showed significant reductions in emotional exhaustion (SMD = −0.752) and depersonalization (SMD = −0.822), but no improvement in personal accomplishment. Interventions effectively reduced key burnout symptoms. |
| **Teng et al., 2023** | Nurses | 4.266 | China | The pooled mean score of mental workload = 68.07%  (CI: 64.39-71.75) | n/s | n/s | n/s | n/s | To determine the levels and influencing factors of mental workload in intensive care unit nurses. It was found a pooled score of 68.07 (95% CI: 64.39–71.75). Factors like demographics, work conditions, and psychology influenced workload. |
| **Huang et al., 2024** | Healthcare workers | 341.014 | China | Prevalence = 47%  (95% CI, 38%-55%) | Prevalence = 38%  (95% CI, 35%-41%) | Prevalence = 34%  (95% CI, 30%-38%) | Prevalence = 26%  (95% CI, 22-29%) | n/s | To systematically evaluate the mental health problems of healthcare workers worldwide during the pandemic and to determine the latest global frequency of COVID-19 associated mental health problems. During the COVID-19 pandemic, 47% of healthcare workers experienced burnout, 38% had anxiety, 34% reported depression, 30% had acute stress, and 26% had PTSD. |
| **Le Huu et al., 2022** | Physicians | 21.939 | France | n/s | n/s | n/s | n/s | The overall standardized pooled mean score was 62.1 [56.2–67.9] (Q=31.0) versus 50.5 [44.8–56.1] (Q =18.3) for studies using six items. For more details, the pooled effort score was 63.8 [53.2–74.4] For the group using the three items version, 61.7 [51.7–71.7] for the four items version, and 62.4 [49.4–75.5] for the five items version. | To estimate the efort–reward imbalance prevalence (ERI) among physicians. It was found that 40.2% of physicians experience ERI. The ERI rate varied significantly, with higher scores in physicians compared to the general workforce. Effort scores showed low variability, while reward scores varied more. |
| **Chen et al., 2022** | Healthcare workers | 18.382 | China | n/s | Prevalence = 43%  (CI: 36-50) | Prevalence = 45%  (CI: 37-52) | n/s | n/s | To systematically review the prevalence of anxiety and depression among frontline healthcare workers during the COVID-19 pandemic. 43% of frontline healthcare workers experienced anxiety and 45% faced depression. Higher prevalence was seen in women, married individuals, those with children, and nurses. Workers with lower education had higher anxiety rates, while those with higher professional titles had more depression. |
| **Colin et al., 2023** | Healthcare workers | 313 | France | Significant impact for emotional exhaustion (P = 0.004), but not for depersonalization or and impairment of personal achievement. | Significant decreased anxiety compared to respective CG. Among the three types of recorded music, the best mean rating score was for Mozart’s music (P < 0.01). | n/s | n/s | n/s | To examine the efficacy of music interventions on stress parameters by selecting studies conducted in genuine care stress conditions. Most studies showed significant benefits, with customized playlists in music therapy (MT) showing advantages over time. Music interventions generally reduce stress, though the article emphasized that more research would be needed to explore the impact of different music types, session frequencies, and long-term effects. |
| **Khatatbeh et al., 2022** | Nurses | 9.859 | Hungary | Negative correlation between nurses’ burnout and their QOL or professional QOL | n/s | n/s | n/s | n/s | To systemically review the relationship between nurses’ burnout and quality of life and to introduce practical recommendations to reduce nurses’ BO and improve their QOL. The review included 21 studies conducted from 2009 to 2021, most of which found significant negative associations between burnout and various aspects of nurses' QOL. |
| **Xia et al., 2023** | Nurses | 1.057 | China | Gender was not a significant predictor of job burnout.   Education level was significantly associated with job burnout.   Nurses with bachelor’s degrees have the highest level of job burnout among all academic groups. | n/s | n/s | n/s | n/s | To synthesize current research on predictors related to burnout affecting Chinese nurses. Key factors found to predict burnout include good working relationships and internally controlled individuals. However, some predictors showed contradictory results, highlighting the complexity of burnout and the challenges in effectively addressing and preventing it. |
| **Zhang et al., 2024** | Healthcare workers | 11.841 | China | n/s | n/s | n/s | Prevalence = 29.2%  (CI: 20.7%-37.7%) | n/s | To estimate the prevalence and influencing factors of post-traumatic stress disorder (PTSD) among Chinese healthcare workers during COVID-19. It was found a PTSD prevalence of 29.2%. Factors protective against PTSD include being female, a nurse, married, working on the front line, having less work experience, having family/friends diagnosed with COVID-19, and having a history of chronic disease. Conversely, higher education, social support, and psychological resilience are also protective factors |
| **Harris et al., 2023** | Healthcare workers | 7.606 | UK | n/s | 46.4% participants in the stalking campaign were significantly more anxious  35.7% reported increased anger  28.6% reported feeling significantly less outgoing | n/s | n/s | n/s | To assess prevalence of stalking, and associated impacts and methods of coping reported by mental health professionals, irrespective of perpetrator type. The search identified 11 peer-reviewed papers, with stalking prevalence rates ranging from 10.2% to 50%, with higher quality studies reporting narrower ranges (13.9%–14.3%). The review found that stalking significantly affected MHPs' confidence and competence at work, prompting lifestyle and workplace changes to mitigate impacts. Interestingly, stalking by colleagues and intimate partners was reported as frequently, if not more, than by clients |
| **Algamdi., 2022** | Nurses | 2.509 | Saudi Arabia | Prevalence = 62.76%  (47.30-77.5) | n/s | n/s | Prevalence = 66.84%  (47.15-83.98) | n/s | To systematically review and comprehensively analyse findings of studies reporting oncology nurses' compassion satisfaction (CS), burnout (BO) and secondary traumatic stress (STS), measured by the professional quality of life (ProQOL) scale. The findings showed the prevalence of CS, BO, and STS at 22.89%, 62.79%, and 66.84%, respectively. No significant correlations were found for independent variables, possibly due to small sample sizes. A weak negative correlation was observed between CS and BO, and a weak positive correlation between CS and STS. |
| **Alberque et al., 2022** | Healthcare workers | 4.842 | France | n/s | n/s | n/s | Prevalence = 14%  (95 CI 10% to 17%) | n/s | This systematic review and meta-analysis aimed to evaluate the prevalence of post-traumatic stress disorder (PTSD) among healthcare workers (HCWs) following the 2003 SARS-CoV-1 epidemic. Fourteen studies involving 4,842 HCWs were included. The overall PTSD prevalence was 14%, with higher rates during the epidemic (16%) and within six months after (19%). PTSD prevalence decreased to 8% one year after the epidemic, with a further decrease to 10% after three years. The findings suggest that PTSD remained prevalent long-term among HCWs following SARS-CoV-1. |
| **de Vargas et al., 2023** | Nurses | 52.270 | Brazil | Prevalence = 52.1% | Prevalence = 55.2% | Prevalence = 58.5% | Prevalence = 65.9% | Prevalence of fear = 52.1% | To assess the prevalence of mental health symptoms in nursing professionals during the COVID 19 pandemic on the American continent. From 7,467 studies, 62 were included, covering 52,270 nursing professionals. The overall prevalence of at least one mental health symptom was 56.3%. The most prevalent symptoms were burnout (52.1%) and fear (52.1%). |
| **You et al., 2022** | Nurses | 7.265 | China | n/s | n/s | n/s | n/s | n/s | To systematically evaluate the status and distribution characteristics of work alienation among nurses in China. The meta-analysis found a moderate level of work alienation (score of 35.43). Factors associated with higher alienation included being male, having higher education levels, low professional titles, being unmarried, having shorter work experience, earning lower salaries, and working in departments like emergency, intensive care, internal medicine, and surgery. |
| **Bekelepi et al., 2022** | Nurses | 296 | South Africa | Mindfulness meditation, relaxation techniques and mindfulness exercises significantly reduced perceived stress and burnout among psychiatric nurses | Mindfulness-based stress reduction programmes have showed impact in reducing the levels of anxiety | Mindfulness-based stress reduction programmes have showed impact in reducing the levels of depression | n/s | n/s | To examine effective stress reduction interventions for nurses and to identify key elements of these successful interventions. Some interventions found were mindfulness-based stress reduction, burnout prevention, communication skills training, and resilience programs. Four key elements were identified as crucial for success: educational support, interpersonal skills, psychological support, and adaptive coping. The findings emphasize the need for diverse interventions to help psychiatric nurses cope with stress, though more research is needed in acute psychiatric settings. |
| **Claponea et al., 2022** | Physicians | 3.071 | Romania | Prevalence = 14.7% to 90.4% | Anxiety and COVID-19-related burnout were significantly associated with the physician’s age, gender, and years in practice. | Burnout was positively associated with a history of depression | n/s | n/s | To assess the prevalence of burnout among physicians working in the healthcare system during the COVID-19 pandemic, and discovering the main factors associated with burnout syndrome among the population of physicians. The prevalence ranged from 14.7% to 90.4%, with higher rates linked to factors such as being female, less experienced, unmarried, and without children. The study identified significant associations between burnout and high levels of anxiety, depression, and stress. The highest burnout rates were observed in physicians from South Korea (90.4%), followed by Saudi Arabia (80.2%) and Ireland (77%). Key contributing factors included the lack of personal protective equipment and organizational health issues. |
| **Chen et al., 2022** | Doctors | 23.595 | China | n/s | n/s | n/s | n/s | n/s | To evaluate the village doctors’ job satisfaction status and to provide guidelines for the healthcare policies. The overall job satisfaction score was 3.19 (out of 5), indicating a "neither satisfied nor dissatisfied" level, which dropped to 2.76 after adjusting for publication bias, signaling dissatisfaction. Key factors contributing to dissatisfaction included financial rewards, job security, and work stress. The study highlighted the need for improvements in the working conditions of rural health workers, especially in low- and middle-income countries. |
| **Sulosaari et al., 2022** | Nurses | 1.009 | Finland | Mindfulness-based programmes seemed to reduce nurses' burnout | n/s | n/s | n/s | n/s | To identify mindfulness-based interventions and outcome measures and to evaluate the effect on the psychological well-being of nurses. Outcome measures included stress, depression, anxiety, burnout, resilience, quality of life, self-compassion, happiness, and mindfulness levels. Ten of the studies showed positive effects of mindfulness interventions on nurses' well-being. The review concluded that mindfulness-based interventions could improve nurses' psychological health. |
| **Yoo et al., 2022** | Nurses | 3.701 | Korea | n/s | n/s | n/s | n/s | n/s | To assess the extent of knowledge of the effectiveness of cognitive‐behavioral therapy (CBT) for healthcare workers by type of delivery. It was found that face-to-face (FTF) CBT interventions were effective for work stress, work-related outcomes, and mental and musculoskeletal problems. Non-face-to-face (NFTF) interventions, however, showed effectiveness for stress and insomnia. While FTF interventions yielded significant results, their impact was not distinctly different from NFTF interventions. |
| **Sheng et al., 2022** | Doctors | 7.595 | China | Pooled burnout rate showed:  37% = high emotional exhaustion (EE) 28% = high depersonalization (DP) 26% = low personal exhaustion (PA) | n/s | n/s | n/s | n/s | To systematically measured the global prevalence of burnout among GPs. The findings showed that 37% of GPs experienced high emotional exhaustion (EE), 28% had high depersonalization (DP), and 26% exhibited low personal accomplishment (PA). Subgroup analyses revealed that high EE and DP rates were more common in studies conducted from 2001 to 2009, while high DP rates were more prevalent in Europe, and low PA rates were associated with high-quality studies. The study highlights the need for health managers to tailor strategies to reduce burnout and improve the well-being of GPs to ensure the retention of this essential workforce in primary healthcare. |
| **Johns et al., 2022** | Doctors | 33.281 | UK | n/s | Pooled prevalence = 25.8%  (95 CI 20.4%-31.5%) | Pooled prevalence = 20.5%  (95 CI 16.0%-25.3%) | n/s | n/s | To analyse the evidence emerging from the first year of the COVID-19 pandemic. The findings revealed that 47% of healthcare workers experienced job burnout, 38% reported anxiety, 34% suffered from depression, 30% had acute stress disorder, and 26% faced post-traumatic stress disorder. Nurses represented the majority (63.6%) of participants, with women accounting for 82.8%. The study emphasized the widespread mental health challenges faced by healthcare workers during the pandemic, with job burnout being the most common issue. Despite the pandemic being controlled globally, its long-term psychological impact on healthcare workers remains significant. |
| **Wang et al., 2024** | Nurses | 35.621 | China | Occupational burnout was found to have a negative impact on nurses' posttraumatic growth | n/s | n/s | n/s | n/s | The aim of this stud was to explore the levels and related factors of post-traumatic growth (PTG) among nurses, based on a systematic review of 55 studies involving 35,621 nurses across 11 countries and regions. The pooled mean score indicated a moderate level of PTG (55.69, 95% CI: 50.67–60.72). The findings suggest that nurses commonly experience PTG after exposure to traumatic events, highlighting individual, work-related, and social/organizational factors as potential determinants. The review emphasizes the importance of addressing these factors in interventions to provide nurses with resources and support to cope with stress and trauma, potentially facilitating PTG. |
| **Bai et al., 2023** | Doctors | 3.610 | China | Pooled prevalence = 61.7%  (95 CI 48.6-73.2%) | n/s | n/s | n/s | n/s | This systematic review and meta-analysis aimed to compare burnout rates among pulmonologists and respiratory therapists before and after the COVID-19 pandemic. The study included 16 studies, with 3610 participants, of which 2336 reported burnout. The pooled prevalence of burnout was 61.7% (95% CI: 48.6–73.2%), with burnout rates during COVID-19 (68.4%) significantly higher than before the pandemic (41.6%) (p = 0.01). Meta-regression analysis showed that the COVID-19 pandemic significantly contributed to the increased burnout rate (p = 0.04). |
| **Long et al., 2023** | Dentists | 6.039 | China | Prevalence = 13%  (95% CI: 6-23) | n/s | n/s | n/s | n/s | To investigate the prevalence of professional burnout among dentists. After reviewing 15 studies involving 6038 participants, the overall burnout prevalence was found to be 13% (95% CI: 6–23%). Subgroup analysis revealed higher burnout rates in Europe and lower rates in the Americas. Additionally, cross-sectional studies reported lower burnout rates compared to longitudinal studies, and burnout prevalence in the last decade was significantly lower than a decade ago. The findings suggest a relatively low but decreasing prevalence of burnout among dentists, emphasizing the need for continued focus on their mental health to prevent and manage burnout effectively. |
| **Ma et al., 2023** | Nurses | 5.304 | China | 36.4% = high levels of EE   28.26% = high levels of DP   28.69% = low levels of PA | n/s | n/s | n/s | n/s | This study had the aim of examining burnout among oncology nurses, finding high levels of emotional exhaustion (36.4%), depersonalization (28.26%), and low personal accomplishment (28.68%). Asian nurses had the highest burnout scores, while nurses in the Americas showed better personal accomplishment. Factors influencing burnout varied by region: personal traits in Asia and work-related factors in Europe. The study highlighted a lack of interventions, particularly in Asia, and emphasized the need for region-specific strategies to support oncology nurses. |
| **Xiong et al., 2022** | Healthcare workers | 65.706 | China | n/s | Prevalence = 17%  (95% CI 13–21%) | Prevalence = 15%  (95% CI 13–16%) | Prevalence = 27%  (95% CI 16–38%) | n/s | The aim of this study was to investigate the psychological impact of COVID-19 on healthcare workers (HCW) in China, focusing on post-traumatic stress symptoms (PTSS), anxiety, depression, sleep disturbances, and profession-related burnout. It included 44 studies with 65,706 HCW participants. The results showed moderate to severe PTSS, anxiety, depression, and sleep disturbances affected 27%, 17%, 15%, and 15% of HCWs, respectively. Mild to severe symptoms were found in 31%, 37%, and 39% for PTSS, anxiety, and depression. Higher prevalence was seen in frontline workers, females, nurses, and those in Wuhan. |
