# Supplementary figures and images for: Strategies to support the mental health and well-being of health and care workforce: a rapid review of reviews
Source: Front Med (Lausanne). 2025 Mar 19;12:1530287. doi: 10.3389/fmed.2025.1530287 (PMC11961965; doi:10.3389/fmed.2025.1530287)

**Supplementary 1**. Search strategy description.


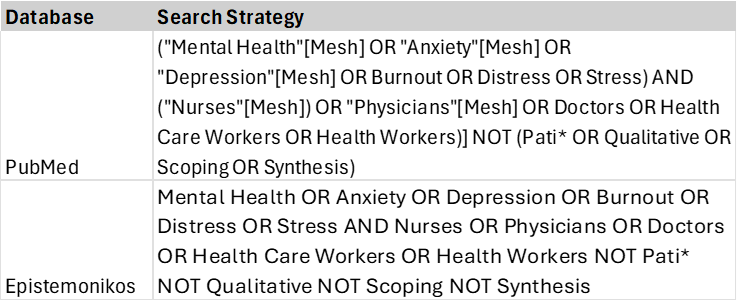

Supplement: Supplementary file 4 [file Supplementary_file_1.docx]
